# Urinary Biomarkers in Cancer: A Scoping Review

**DOI:** 10.3390/cancers18203328

**Published:** 2026-10-15

**Authors:** Evelyn Kurotova, Jayni Shah, Lucia Pueyo Valles, Edwina Burke, Ruben Berreby, Yong-Jie Lu, Silvana Debernardi, Tatjana Crnogorac-Jurcevic

**Affiliations:** Centre for Cancer Biomarkers and Biotherapeutics, Barts Cancer Institute, Queen Mary University of London, London EC1M 6BQ, UK

**Keywords:** urine, biomarkers, cancer

## Abstract

Cancer remains a major global health challenge and a leading cause of mortality worldwide. As incidence continues to rise, early detection remains one of the most effective strategies for improving survival and reducing disease burden. Urine is an attractive source of biomarkers due to its non-invasive collection, ease of repeated sampling, and rich molecular composition. This scoping review evaluates the current landscape of urinary biomarkers for the early detection of cancer and their potential for clinical translation. A detailed literature search was conducted using PubMed, Embase, and Web of Science to identify studies investigating urinary biomarkers for cancer detection. Biomarkers were categorised by analyte class, and diagnostic performance, validation status, and clinical applicability were assessed. Urinary biomarkers demonstrated diagnostic potential across multiple malignancies, including lung, breast, pancreatic, hepatobiliary, colorectal, renal and ovarian cancers, while several urinary assays are already clinically available for bladder and prostate cancers. Promising diagnostic accuracy was reported for multiple biomarker classes, particularly multimarker panels integrating metabolites, proteins, nucleic acids, and epigenetic signatures. However, most studies were exploratory, involving small cohorts, methodological heterogeneity, and limited independent validation. Urinary biomarkers represent a promising and rapidly evolving approach to non-invasive cancer detection. Despite encouraging diagnostic performance, few candidates have progressed to clinical implementation. Large prospective multicentre studies, rigorous validation, and assay standardisation are essential to establish urinary biomarkers as reliable tools for cancer screening, early diagnosis, and risk stratification.

## Simple Summary

Cancer remains a major global health challenge; however, survival improves substantially when the disease is detected early. Urine represents an attractive diagnostic biofluid because it can be collected easily, non-invasively, and repeatedly. This scoping review evaluated published studies investigating urinary biomarkers for the early detection of multiple cancer types. Urine was found to contain diverse biomarker classes, including metabolites, proteins, DNA and RNA, which can distinguish cancer patients from those without disease. Several urinary biomarkers demonstrated promising diagnostic performance; however, most studies were conducted in relatively small cohorts and exhibited considerable heterogeneity in study design, populations, and analytical approaches. While the current evidence supports the potential of urine-based biomarkers as a convenient and patient-friendly approach for cancer detection, further large-scale, prospective, and well-designed validation studies are needed before these biomarkers can be considered for routine clinical implementation.

## 1. Introduction

### 1.1. Importance of Early Detection

Cancer remains one of the greatest global health challenges, with nearly 20 million new cases diagnosed annually worldwide, a figure projected to exceed 35 million by 2050. The disease is responsible for almost 10 million deaths each year, making it the second leading cause of mortality globally [1]. Despite significant advances in prevention, diagnosis, and treatment across multiple tumour types, cancer incidence continues to rise, not only among ageing populations but also increasingly in adults younger than 50 years [2,3]. There is substantial evidence that earlier diagnosis improves patient outcomes and survival, underscoring the need for effective strategies that enable cancer detection at a stage when curative intervention remains possible. In addition, earlier diagnosis not only increases the likelihood of successful treatment but also has the potential to reduce healthcare costs associated with advanced disease management [4].

The impact of stage at diagnosis is particularly evident in cancers that typically present with late-stage and/or non-specific symptoms, such as pancreatic and lung cancer. Because these malignancies are often diagnosed after regional or metastatic spread has occurred, most patients become ineligible for curative treatment, resulting in poor overall 5-year survival rates [5]. A similar relationship between stage and outcome is observed in ovarian cancer, where 5-year survival exceeds 70–95% when the disease is detected at an early, localised stage but decreases dramatically to 10–40% following advanced-stage diagnosis [6]. These marked differences in survival emphasise that earlier detection can provide a critical window for therapeutic intervention before extensive disease progression occurs.

Consequently, there is an urgent need for reliable biomarkers capable of detecting cancer before symptom onset and independently of clinical presentation. Such biomarkers could facilitate a shift towards earlier-stage diagnosis, increasing the proportion of patients eligible for curative treatment and ultimately improving survival outcomes [7].

### 1.2. Biological Rationale and Advantages of Urine as a Biofluid for Cancer Detection

Unlike tissue biopsies, which provide a snapshot of a specific tumour region, liquid biopsy biomarkers can capture molecular alterations occurring across the entire tumour landscape, thereby offering a more comprehensive representation of tumour heterogeneity [8]. To date, most liquid biopsy studies have focused on blood-based biomarkers; however, urine represents an attractive, completely non-invasive alternative. Although urinalysis has been used in clinical practice for centuries [9] its potential as a source of cancer biomarkers has only recently gained significant attention.

Compared with blood collection and tissue sampling, urine collection is simple, rapid, non-invasive, and readily amenable to self-collection in a home setting. In addition, repeated sampling is straightforward, facilitating longitudinal monitoring, and large sample volumes can be obtained when required. Importantly, as a waste product, urine is not subject to the same tight homeostatic regulation as blood. As a result, early molecular changes associated with cancer development may be masked in the circulation but could become concentrated and more readily detectable in urine [10].

Urine contains a diverse range of analytes with biomarker potential. These biomarkers may originate from circulating blood components released by tumour cells through apoptosis, necrosis, or active secretion and subsequently cleared through renal filtration. Alternatively, they may arise from direct shedding, secretion, or excretion by tumour cells within the urogenital tract [11]. As a result, urinary biomarker alterations are not restricted to genitourinary malignancies but have also been reported across a wide range of non-urological cancers. Biomarker classes identified in urine include microRNAs (miRNAs), circulating tumour DNA (ctDNA), extracellular vesicles (EVs), proteins, and metabolites [12,13,14]. Importantly, recent mechanistic evidence from Kawaguchi et al. demonstrated active glomerular transcytosis of circulating tumour-derived small extracellular vesicles into urine, strengthening the biological rationale for urine-based liquid biopsy [15]. These characteristics highlight urine as a versatile and highly promising biofluid for cancer detection. The studies reviewed here demonstrate the breadth of urinary biomarker applications across multiple tumour types and underscore their potential role in improving early cancer detection.

## 2. Methods

For this scoping review, PubMed, Embase, and Web of Science were searched for studies investigating urinary biomarkers for the early detection of 11 common cancer types (lung, breast, gastric, hepatocellular, cholangiocarcinoma, pancreatic, colorectal, renal, ovarian, ampullary and duodenal), published between January 2019 and February 2025. The selected time frame was chosen to capture the most recent developments in this rapidly evolving field, while maintaining sufficient overlap with previous reviews to assess the consistency and completeness of the search strategy. Although earlier literature was initially considered, many studies were limited by small sample sizes, methodological shortcomings and inconsistent reporting. Consequently, the review focused on more contemporary research which enabled a more robust assessment of clinically relevant urinary biomarkers while also capturing previously identified candidates that have subsequently undergone further validation.

Consistent with the methodology for a scoping review, a protocol was not prospectively registered or published. No formal assessment of methodological quality or risk of bias was conducted for the included studies. The PRISMA-ScR checklist can be found in the Supplementary Table S1.

**Figure 1:**
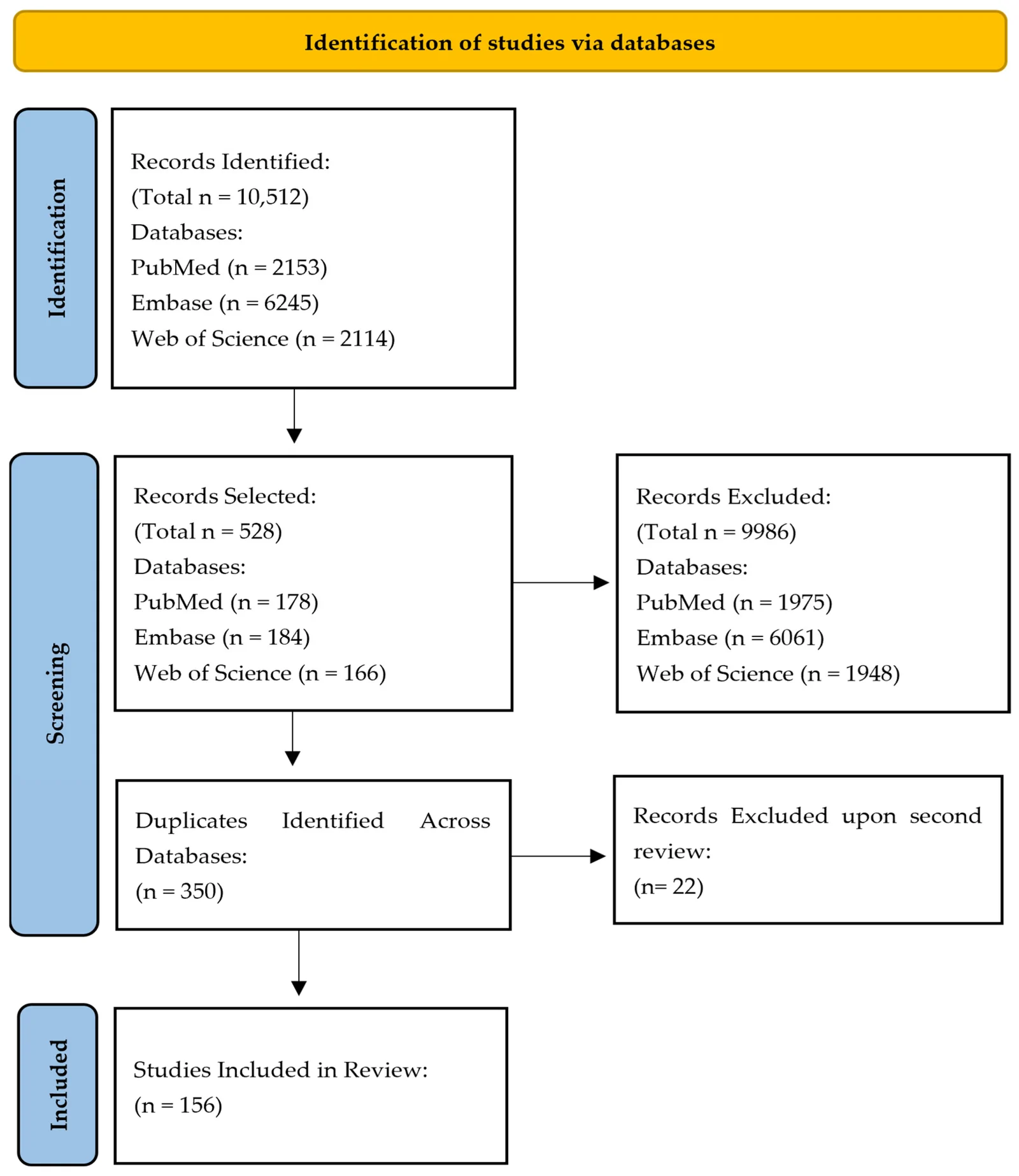
PRISMA flow diagram outlining the methodology of the review.

Searches were conducted using combinations of the following keywords and Medical Subject Headings (MeSH) terms: (urine) OR (urin*) AND (biomarker) OR (marker) OR (non-invasive) OR (noninvasive) AND (detection) OR (early detection) OR (diagnosis) OR (early diagnosis) OR (diagnos*) AND (tumour) OR (tumor) OR (adenocarcinoma) OR (cancer) OR (carcinoma) OR (neoplasm) AND (cancer type). Searches were limited to English-language publications and human studies. Studies were excluded if they did not utilise urine samples, involved non-human models, were unrelated to cancer, or did not report diagnostic performance measures, including area under the receiver operating characteristic curve (AUC), sensitivity (SN), and specificity (SP). However, studies reporting at least one of these parameters and providing comprehensive biomarker evaluation were considered for inclusion. Titles and abstracts were screened for relevance, and non-eligible publications were excluded. Reviews, case studies, conference abstracts, and publications describing established clinical practice or treatment guidelines were also excluded. The remaining articles underwent full-text evaluation, and eligibility was confirmed according to the predefined inclusion and exclusion criteria. Duplicate records were subsequently identified and removed. To render the review more comprehensive, backward citation searching of eligible articles and relevant review papers was also performed. This process identified a small number of further studies that were not captured by the initial database searches and were therefore included in the final analysis. The overall study selection process is summarised in Figure 1.

Bladder and prostate cancers were considered separately because they were found to have an extensive literature base, including several urinary biomarker assays that are already used in clinical practice. An alternate search was completed for these two pathologies, compiling a list of already available urinary tests.

## 3. Results

**Figure 2:**
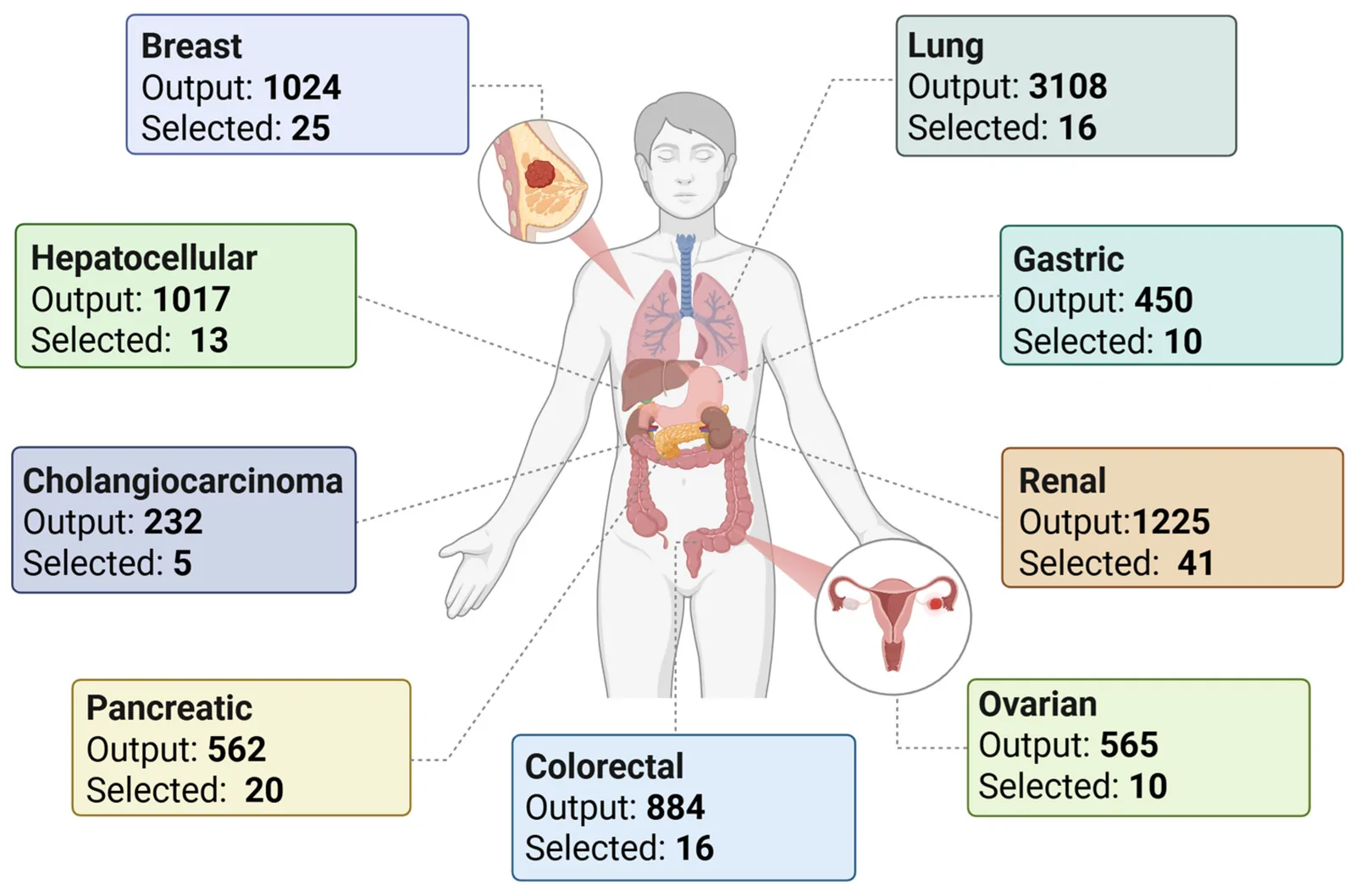
Total number of search results (output) across all databases, and the final number of selected relevant publications for nine different cancer types. Created in BioRender. Kurotova, E. (2026) https://BioRender.com/600vvcp.

The literature search identified over 10,000 papers describing novel urinary biomarkers for the detection of 11 cancers, of which 156 publications were deemed eligible (Figure 1 and Figure 2). The distribution of records across the three databases is presented in Table 1. The search for Ampullary and Duodenal cancers retrieved 20 and 1425 records respectively; however, none fulfilled the predefined eligibility criteria and were therefore excluded from the output summary in Figure 2.

As illustrated in Figure 2 and Table 1, the literature search yielded a substantial number of records despite the use of comprehensive search strategies and specific MeSH terms. The highest numbers of records were identified for lung (*n* = 3108), renal (*n* = 1225), breast (*n* = 1024) and hepatocellular (*n* = 1017) cancers. Unexpectedly, many of the retrieved publications investigated plasma biomarkers rather than urinary biomarkers, notwithstanding the use of urine-focused search criteria. Furthermore, a large proportion of studies did not report clinically relevant diagnostic performance measures, including SN, SP and AUC, as they were often preliminary investigations conducted in relatively small cohorts. Similarly, the majority of the 156 relevant studies predominantly used healthy controls, with only 52 specifying the use of benign controls (33%). There was limited discussion of the presence and the potential impact of comorbidities on urinary biomarker performance. The use of validation cohorts was also limited to only 37 papers (24%).

Across the studies considered, methods of urine collection and processing differed vastly. It is not possible to report any findings pertaining to the normalisation of urine samples within our literature search, as this information is not readily available. Many papers do not disclose this information; therefore, it is unclear whether urine normalisation did not take place or whether it was not specified in the text. Storage conditions were described more frequently, with most studies storing urine samples at −80 °C.

**Figure 3:**
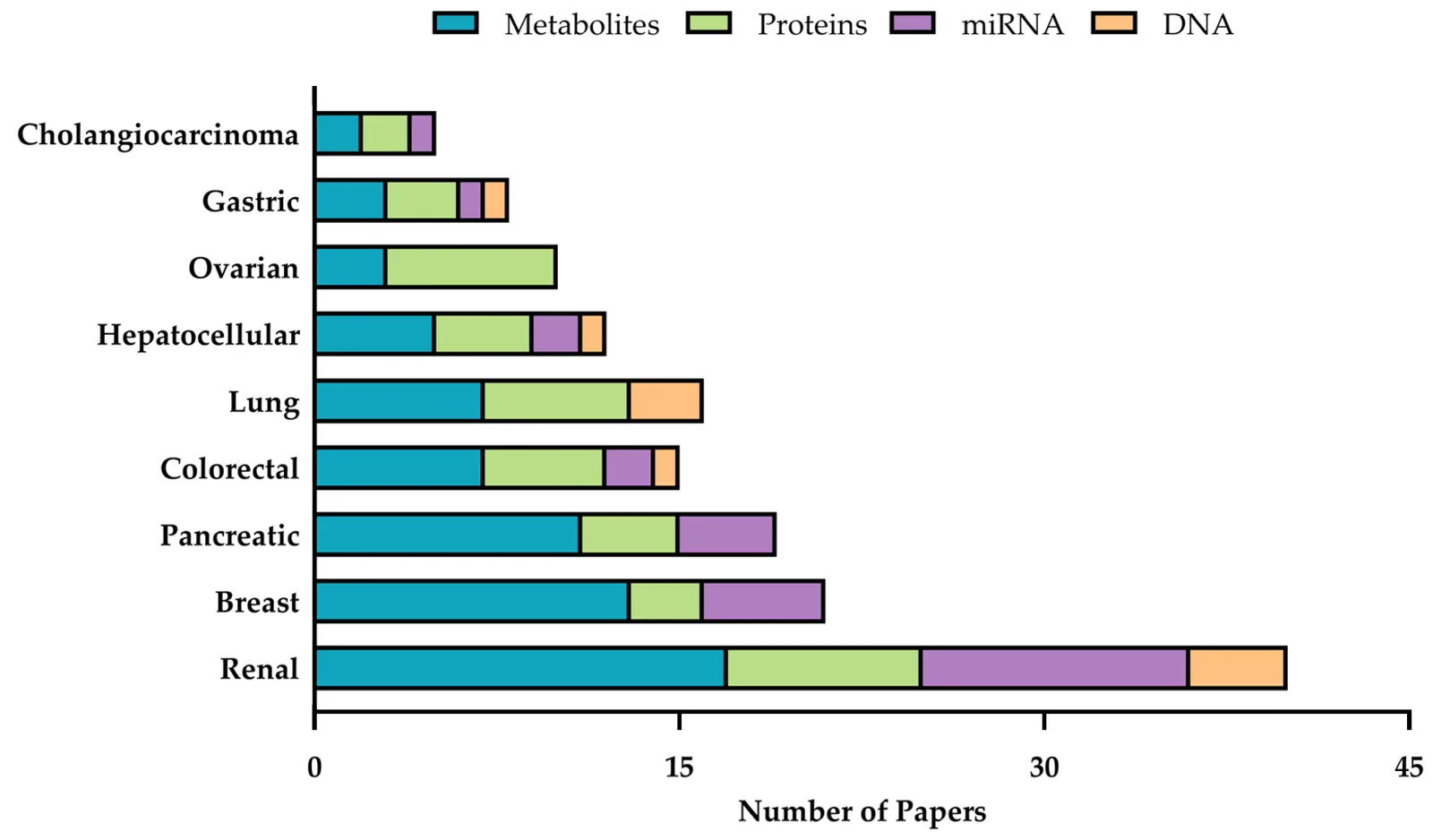
Distribution of the four most common analyte groups across nine cancers.

A number of different analytes were identified in the selected publications. The most commonly found are shown in Figure 3, and can be classed into four categories: Metabolites, Proteins, miRNA and DNA.

Metabolites, including volatile organic compounds (VOCs), were most frequently studied across all nine cancers, being reported in 68 manuscripts, thus contributing to over 40% of the results. The majority of these studies described various metabolites in different combinations to establish potential panels for cancer detection; however, most findings were preliminary. Notably, among the 68 metabolite studies, only 25 papers (37%) specified the use of benign controls, while only 14 papers (21%) included a validation cohort in their experiments. Proteins were also commonly studied (42 papers, 27%), both individually and as panels. Additionally, miRNAs were described in 26 papers (17%), and DNA in 10 papers (6%), seven of which reported on DNA methylation markers. A small number of additional analytes were also identified, such as steroids in breast cancer and metal elements in pancreatic cancer [16,17].

Detailed findings of the analytes found in each cancer type included in Table 1 are described in the following sections.

### 3.1. Lung Cancer

Lung cancer remains the leading cause of cancer-related mortality worldwide. Approximately 75% of patients are diagnosed with advanced-stage disease (stage III/IV), and despite substantial advances in treatment, outcomes remain poor [18]. In the UK, patients diagnosed with stage IV disease have a 1-year survival rate of only 15–19%, compared with 81–85% for those diagnosed at stage I [19]. Currently, low-dose computed tomography (LDCT) is the only approved detection modality, highlighting the need for accurate, non-invasive biomarkers for early detection and risk stratification [20].

Several classes of urinary biomarkers have shown promise for lung cancer detection (Table 2), including metabolites, proteins, cfDNA methylation markers, extracellular vesicles (EVs), and VOCs. Metabolomic signatures identified by gas chromatography and UPLC-MS achieved AUCs of up to 0.88 [21], while individual metabolites such as O-aminohippuric acid and 4-methoxyphenylacetic acid (4MPLA) demonstrated high diagnostic accuracy (AUCs 0.837–0.85; SN up to 82.1%; SP up to 88.2%) [22,23]. A further study of 10 metabolites by Jia et al. showed promising AUCs of 0.94 in the training set when comparing LC and benign controls (pneumonia samples). In the test set, the same value (0.94) was obtained when comparing LC to both healthy and benign samples [24].

Urinary EV biomarkers showed particularly strong performance, with AUCs reaching 0.96 [25]. The combination of urinary HE4 and CEA showed an increased sensitivity when distinguishing LC from healthy controls (combined, 95%; HE4, 72.38% and CEA, 60.65%); however, this was not the case with specificity, which showed a decrease in the combined panel (combined, 75.7%; HE4, 94% and CEA, 86%) [26].

Similarly, urinary cfDNA methylation markers (CDO1, SOX17, TAC1, and HOXA9), protein biomarkers (PLG, FGG, and MDH2), and mutation-based assays, including LINE1 and EGFR, demonstrated encouraging diagnostic and molecular profiling potential [27,28,29,30,31]. VOC-based signatures (AUC 0.91; SN 85%; SP 90%) and exosomal markers such as WASL, STK10, and WNK1 further expand the range of promising urinary biomarkers [32,33]. Collectively, these studies support the feasibility of urine-based testing for lung cancer detection; however, large multicentre validation studies are needed before their clinical implementation.

**Table 2:** Urinary biomarkers in lung cancer (LC).

Biomarker Type	Biomarker(s)	Study Population	Method	AUC (95% CI)	SN (95% CI)	SP (95% CI)	Reference
Metabolites	17 metabolites	548 LC, 563 BeC, 229 H	GC, UPLC-MS	0.88	NR	NR	[21]
Metabolites	EVS: Kanzonol Z, Xanthosine, Nervonyl carnitine, 3,4-Dihydroxybenzaldehyde	75 LC, 27 H	LC-MS/MS	0.96	NR	NR	[25]
Metabolites	O-aminohippuric acid	46 LC, 185 H	LC-MS/MS	0.83	NR	NR	[22]
DNA Methylates	CDO1, TAC1, HOXA9, SOX17	74 NSCLC, 27 H	qMSP	Combined: 0.70	73	92	[27]
Metabolites	Creatine riboside, N-acetylneuraminic acid	Training: 375 LC, 471 H Validation: 285 LC, 220 H	UPLC-MS/MS	Non-smokers exploratory cohort: 0.94	NR	NR	[34]
Metabolites	10 metabolites	77 LC (including 29 LUAD and 25 LUSC), 33 LC are from initial visit (IV); 33 PN; 63 H	Titanate-based perovskite material Rh-doped SrTiO3 (STO/Rh)-assisted LDI-MS + machine learning (NN)	NN model: Training set, LC v H, 0.94; LC v PN: 0.94; LC IV v H, 0.95. Test set, LC v H 0.94, LC v PN 0.86. LC v PN + H 0.94	Training set: LC IV v H, TPR: 86.46	Training set: LC LC IVv H, TNR: 93.75	[24]
Protein	HE4, CEA, NSE, CYFRA 21-1, and ProGRP	212 LC (28 LUSC, 136 LUAD, 14 ASC, 29 SCLC, 3 LCNEC, 2 NSCLC) (42 stage I/II, 170 stage III/IV); 80 BeC, 100 H	HE4, Pro-GRP, and CEA: CMIA and architect i4000 analyzer; CYFRA 21-1 and NSE: ECLIA and Cobas e602 analyzer	HE4: LC v H, 0.87 (0.83–0.91); LC v BeC, 0.83 (0.78–0.87); LC v BeC v H, 0.85 (0.81–0.89); CEA: LC v H, 0.76 (0.71–0.82); Combined: LC v H, 0.89 (0.85–0.93)	HE4: LC v H, 72.38; CEA: LC v H, 60.65; Combined: LC v H, 95	HE4: LC v H, 94; CEA: LC v H, 86; Combined: LC v H, 75.7	[26]
Proteins (exosomal)	WASL, STK10, WNK1	44 LC, 30 H	MS, ELISA	Combined: 0.76	NR	NR	[33]
DNA methylates	CDO1 and SOX17 (combined)	46 NSCLC, 50 H	qMSP	0.78	57	86	[30]
Protein	MDH2	1091 NSCLC, 736 H	ELISA	0.77	70.13	66.11	[28]
Metabolite	4MPLA	21 LC, 14 H	1H NMR	NR	82.10	88.20	[23]
Proteins	PLG, FGG	112 NSCLC, 197 H	ELISA	Combined: 0.927	77.68	94.55	[31]
DNA	LINE1-97, 266 bp cfDNA	55 NSCLC, 35 H	qPCR	Stage I/IV, 0.65; Stage III/IV, 0.80	Stage I/IV, 60; Stage III/IV, 62	Stage I/IV, 60; Stage III/IV, 89	[29]
VOCs	e 2-pentanone, 2-hexenal, 2-hexen-1-ol, hept-4-en-2-ol, 2-heptanone, 3-octen-2-one, 4-methylpentanol, 4-methyl-octane	46 LC Stage I/III, 81 H	GC-IMS	0.91	85	90	[32]
Proteins	KNG1, OPN AAT	31 LUSC (13 Stage I/II, 18 Stage III/IV), 20 BeC	ELISA	KNG1 0.81; OPN 0.75; AAT 0.86	KNG1 90; OPN 85; AAT 100	KNG1 59; OPN 65; AAT 53	[35]
Proteins	FGG, GPI and CDK1	72 SCLC, 72 H	MALDI-TOF-MS	≥0.80	85	80	[36]

ASC, adenosquamous carcinoma; H, healthy; LCNEC, large cell neuroendocrine carcinoma; LUAD, lung adenocarcinoma; LUSC, lung squamous cell carcinoma; NN, neural network; NR, not recorded; NSCLC, non-small cell lung cancer; PN, pneumonia patients; SCLC, small cell lung cancer; TNR, true negative rate; TPR, true positive rate.

### 3.2. Breast Cancer

Breast cancer (BC) is one of the most common malignancies worldwide and the most frequently diagnosed cancer in the UK, accounting for 58,756 new cases in 2022 [37]. Although mammography is an established screening tool, its performance is reduced in women with dense breast tissue, and participation remains suboptimal due to barriers such as appointment inconvenience and fear of, or prior experience with, pain [38,39]. Consequently, there is considerable interest in developing non-invasive alternatives or complementary approaches for early detection, and urine is a viable biospecimen for this purpose.

The search identified 25 studies investigating urinary biomarkers for BC detection (Table 3). Notably, three studies evaluated the N-Nose detection platform, which uses the nematode *Caenorhabditis elegans* (*C. elegans*) and is widely adopted in Japan. The assay demonstrated promising diagnostic performance, with SNs ranging between 73.4% and 98% [40,41,42,43]. Further VOC studies such as the one from Kure et al., reported that urinary 2-propanol and 2-butanone complemented existing detection methods, achieving high SN and SP in both training (93.3% and 83.3%) and validation (84.0% and 90.5%) cohorts [44]. The 10-fold cross-validation of the statistically significant panel of VOCs showed an AUC of 0.88, compared to 0.97 in the discovery set [45].

A multi-platform volatile and non-volatile metabolite signature identified using HILIC-MS, RPLC-MS, and GC-HRMS achieved a SN of 86.4% and SP of 89.2%, although further validation in larger cohorts is required before clinical translation [46]. In fact, most metabolite studies remain in the discovery phase, and are limited by relatively small sample sizes, and often use healthy rather than benign controls, potentially inflating reported diagnostic performance. Nevertheless, the growing body of evidence highlights the significant potential of urinary biomarkers as a convenient and non-invasive approach for breast cancer detection, warranting further validation in large, independent cohorts.

**Table 3:** Urinary biomarkers in breast cancer (BC).

Biomarker Type	Biomarker(s)	Study Population	Method	AUC (95% CI)	SN (95% CI)	SP (95% CI)	Reference
*C. elegans* VOCs	VOCs	128 BC (7 Stage 0; 57 Stage I; 36 Stage II; 13 Stage III; 15 Stage IV)	N-NOSE	NR	73.40 overall (Stage 0: 57.1; Stage I, 68.4; Stage II, 77.8; Stage III, 84.6; Stage IV, 80.0)	NR	[40]
Modified nucleosides	Modified nucleosides	43 BC, 50 GC, 48 H	AC, SERS	0.91	76.70	87.5	[47]
VOCs	2-propanol and 2-butanone	Training: 60 BC, 60 H Validation: 50 BC, 117 H	GC-MS	Training: 0.94; Validation: 0.92	Training: 93.30; Validation: 84	Training: 83.3; Validation: 90.5	[44]
*C. elegans* VOCs	VOCs	10 BC, 143 H	N-NOSE	10× dilution: 0.92; 100× dilution: 0.90	10× dilution: 78.1; 100× dilution: 75; Combination 87.5	10× dilution: 93.7; 100× dilution: 95.1; Combination 90.2	[41]
VOCs	Best of nine possible VOCs: acrolein, 2-pentanone, and methyl allyl sulfide	24 BC, 27 H	HPPI-TOFMS, Mann–Whitney U test	Discovery set: 0.97; 10-fold cross-validation: 0.88	Discovery set: 92.6; 10-fold cross-validation: 85.2	Discovery set: 91.7; 10-fold cross-validation: 83.3	[45]
Glycoprotein	ALCAM	81 BC, 81 H	ELISA	0.95	88.89	89.19	[48]
Metabolites	128 metabolites (115 in urine)	30 BC, 40 H	NMR and GC–qMS	glutamine, betaine and α-hydoxybutyrate: 0.93 (0.86–0.99)	NR	NR	[49]
Various molecules	229 urine molecules (136 urine only)	35 BC, 15 H	UHPLC–QTOF-ESI + MS	>0.80 for 21 urine molecules	NR	NR	[50]
*C. elegans* VOCs	VOCs	36 BC, 36 H	N-NOSE	NR	75	97.22	[43]
RNA methylation	Modified nucleosides (A, Am, m1A, m6A, m6Am, and m62A)	18 BC, 18 H	HILIC-MS/MS	AUC m^6^A, A_m_, m^1^A, and m^6^A_m_: 0.86, 0.71, 0.71, and 0.70, respectively	NR	NR	[51]
VOCs	134 VOCs (11 chemical classes)	80 BC, 88 H	HS-SPME combined with GC-HRMS	Training: 0.91 Validation: 0.88	Training: 76.3 Validation: 76.0	Training: 85.4 Validation: 92.3	[52]
Proteins (ECV)	ECM1, ANXA1	128 BC, 25 BBN, 25 H	proteomic analysis	ECM1: BC v H 0.96; BC v BBN 0.89; ANXA1: BC v H 0.97; BC v BBN 0.85; Combined: BC v H 0.99; BC v BBN 0.94	ECM1: BC v H 79.17, BC v BBN 80.56; ANXA1: BC v H 93.06; BC v BBN 86.11; Combined: BC v H 95.83, BC v BBN 91.67	ECM1: BC v H 100; BC v BBN 100; ANXA1: BC v H 100; BC v BBN 82.61; Combined: BC v H 100; BC v BBN 100	[53]
Steroids	20 (5 selected: testosterone-S, alpha-estradiol, 4-methoxyestradiol, DHEA-S, and epitestosterone-S)	185 BC, 65 H	UHPLC + RF	RF AUC: 0.94; ROC AUC: 0.97	NR	NR	[16]
VOMs	53 VOMs	30 BC, 41 H	HS-SPME, GC–qMS	Top 4 metabolites: 0.84	NR	NR	[54]
Spectra	Biomolecules	43 BC, 50 GC, 48 H,	SERS	BC vs. H: 0.97	BC vs. H: 96	BC vs. H: 93.8	[55]
Modified metabolites	7-methylguanosine, N4-acetylcytidine, dihyroxy-1H-indole glucuronide I, indole-3-acetic acid-O-glucuronide	17 BC, 19 H	LC–MS	0.84, 0.82, 0.78, and 0.83, respectively	NR	NR	[56]
Exosomal miRNA and protein	miR-21, MMP-1/CD63	22 BC, 26 H	qPCR, WB	miR-21: 0.76; MMP-1/CD63: 0.83	miR-21: 71; MMP-1/CD63: 79; Combined: 95	miR-21: 78; MMP-1/CD63: 84; Combined: 79	[57]
miRNA	4039 Total number, 275 filtered miRNAs	32 BC, 50 H	miRNA Seq, qPCR	RF Mean Ca vs. H: Total 0.58; Filtered 0.67	NR	NR	[58]
Metabolites	Volatile and Non-volatile metabolites	66 BC, 49 H	UPLC-Q-Exactive Orbitrap-MS, HS-SPME/GC-MS, RPLC, HILIC	HILIC-MS, RPLC-MS and GC-HRMS fusion: 0.97	HILIC-MS, RPLC-MS and GC-HRMS fusion: 86.37	HILIC-MS, RPLC-MS and GC-HRMS fusion: 89.19	[46]
RNA methylation	Modified nucelosides (A_m_, m^6^A, m^1^A, m^6^A_m_, G_m_, m^1^G, C_m_, m^5^C, U_m_ and m^5^U)	128 EBC, 91 LABC, 109 H,	HILIC–MS/MS with stable isotope dilution	0.78	NR	NR	[59]
Metabolites	1-MA, 1-MG, 8-OHdG + CA15-3	167 BC, 47 BeC, 52 H	Elecsys 2010 System, GC/MS	0.95	Combined: 91.5	Combined: 87.1	[60]
*C. elegans* VOCs	VOCs	24 BC, 18 CRC, 53 H	N-NOSE	PPV: 53; NPV: 97 (Test overall: PPV 67; NPV 97; overall detection rate: 78)	BC: 96, (test overall: 98)	BC: 62 (test overall: 62)	[42]
Metabolites	36 metabolites	40 BC, 38 H	^1^H-NMR	creatine, glycine, trimethylamine N-oxide, serine: 0.90 (0.81–0.99)	NR	NR	[61]
Exosomal miRNAs	miR-17, miR-107, miR-125b, miR-194, miR-222, miR-423, miR-424, miR-660, let7-a, let7-d, let7-e, let7-f, let7-i	69 BC, 40 H	RT-qPCR	miR-424: 0.88; let7-i: 0.86; miR-423: 0.86; let7-f: 0.85; miR-660: 0.85. Fitted model: 0.90–1 (median 0.99)	miR-424 + miR-423 + miR-660 + let7-i: 98.6	miR-424 + miR-423 + miR-660 + let7-i: 100	[62]
Proteins	*m*/*z* 1046.5, 1062.5, 1237.7 and 1727.9 (and others)	50 BC, 50 H	1D SDS-PAGE, MALDI-TOF MS	*m*/*z* 1046.5 + 1062.5 + 1237.7 + 1727.9: 0.99 (0.96–1)	*m*/*z* 1046.5 + 1062.5 + 1237.7 + 1727.9: 88	*m*/*z* 1046.5 + 1062.5 + 1237.7 + 1727.9: 98	[63]

BeC, benign control; BBN, benign breast nodules; EBC, early-stage breast cancer; LABC, late-stage breast cancer; GC, gastric cancer; H, healthy; 1-MA, 1-methyladenosine; 1-MG, 1-methylguanosine; NR, not recorded; 8-OHdG, 8-hydroxy-20 de-oxyguanosine; RF, random forest; VOM, volatile organic metabolite.

### 3.3. Gastric Cancer

Gastric cancer (GC) remains a major global health burden, with almost 1 million new cases diagnosed in 2022, accounting for 4.8% of all cancers, and approximately 660,000 deaths, representing 6.8% of all cancer-related mortality worldwide. GC is currently the fifth most frequently diagnosed cancer and the fifth leading cause of cancer death globally [1,64]. The disease is particularly prevalent in East Asia, especially in China, Japan, and South Korea, where more than 70% of cases are attributable to chronic *Helicobacter pylori* infection. Although age-standardised incidence rates have declined in several countries over recent decades, the absolute number of new GC cases is projected to rise due to population growth and increasing life expectancy [1,64].

The implementation of organised screening programmes has demonstrated the substantial impact of early detection on GC outcomes. Nationwide radiographic and endoscopic screening programmes in Japan and the Republic of Korea have resulted in a shift towards diagnosis at earlier stages and improved patient survival [65,66]. Similarly, regional endoscopic screening initiatives in China have shown promising results and are increasingly being expanded into broader population-based programmes [67,68]. These efforts have contributed to significant improvements in the stage distribution and survival of GC patients, further underscoring the clinical value of early detection strategies [69].

Current risk stratification approaches largely rely on *H. pylori* testing and serum pepsinogen measurements; however, additional non-invasive biomarkers are needed to improve identification of individuals at greatest risk and to facilitate more effective screening programmes. In this context, urine represents a particularly attractive biofluid. Our literature search identified several promising urinary biomarkers across different molecular classes that may complement existing risk-stratification strategies and support earlier detection of disease (Table 4). Among miRNA-based approaches, a urinary panel comprising miR-6807-5p, miR-6856-5p and *H. pylori* status achieved an AUC of 0.89, with 76.9% SN and 88.9% [70]. Protein biomarkers have also shown encouraging results, particularly a panel incorporating TFF1, ADAM12 or BARD1 together with *H. pylori* status, which achieved AUCs of 0.86–0.89 and SN exceeding 90% for stage I GC [71]. However, the strongest performance has been reported for urinary metabolomic signatures. A panel of β-(Pyrazol-1-yl)-L-alanine, D-serine and D-isoleucine, combined with age, achieved an AUC of 0.977 in distinguishing GC patients from healthy controls [72]. Similarly, a urinary metabolite signature comprising lactic acid, 1-methylnicotinamide, glutamine, myo-inositol and 3-indoxylsulfate yielded AUCs of 0.952 and 0.961 in training and validation cohorts, respectively [73]. Notably, an NMR-based metabolomic panel of nine metabolites demonstrated 93.9% SN and 94.1% SP, highlighting the potential of urine metabolomics for early GC detection [74]. Collectively, these findings suggest that urinary metabolite panels currently represent the most promising non-invasive biomarkers for GC, although large prospective validation studies are still required before clinical implementation.

### 3.4. Hepatocellular Carcinoma

Liver cancer remains a major global health burden, with approximately 865,000 new cases and 758,000 deaths worldwide in 2022, making it one of the leading causes of cancer mortality globally [1]. Hepatocellular carcinoma (HCC) accounts for 75–85% of liver cancers and occurs most frequently in East and Southeast Asia and parts of Africa. In the UK, around 6200 people are diagnosed annually, with incidence increasing by approximately 50% over the past decade. Five-year survival remains poor (∼13%), largely owing to late diagnosis [81].

The major risk factors for HCC are chronic hepatitis B virus (HBV) and hepatitis C virus (HCV) infection, together with cirrhosis, although metabolic dysfunction-associated steatotic liver disease (MASLD), non-alcoholic steatohepatitis (NASH), and excessive alcohol consumption are becoming increasingly important contributors with viral hepatitis prevention and treatment improvement [1,82,83,84]. Early detection is critical, as 5-year survival can increase from approximately 18% to 60–70% when tumours are diagnosed at a curable stage [85]. Although surveillance with 6-monthly imaging reduces HCC mortality and increases detection of early-stage disease, implementation remains suboptimal [86,87,88]. Furthermore, the only routinely used biomarker, serum alpha-fetoprotein (AFP), has limited sensitivity, particularly for early-stage disease [89], highlighting the need for improved non-invasive surveillance tools.

Several urinary biomarker studies have shown promise (Table 5). Among metabolomics approaches, a large study of 581 samples demonstrated that matrix-assisted metabolic phenotyping could distinguish healthy controls, high-risk individuals and HCC patients with AUCs exceeding 0.95 in both discovery and validation cohorts, representing the strongest performance reported to date [90]. Other promising biomarkers include urinary NGAL, which differentiated HCC from cirrhosis with 90% SN and 87.5% SP, increasing to 95% SN and 100% SP when combined with serum AFP [91]. Urinary miR-93-5p, measured by RT-qPCR, identified HBV-related HCC with 87.5% SN and 95.4% SP [92]. In addition, a urinary ctDNA panel comprising TP53 249T mutation and methylated RASSF1A and GSTP1, combined with AFP, achieved an AUC of 0.91, with 79.6% SN for all-stage disease and 92% SN for stage 0 HCC [93]. Collectively, these studies highlight the considerable potential of urinary biomarkers for HCC surveillance and early detection, although prospective validation in large at-risk populations remains essential.

### 3.5. Cholangiocarcinoma

Cholangiocarcinoma (CCA) is a relatively uncommon cancer, arising from the biliary epithelium, and accounts for approximately 15% of primary liver malignancies [103,104,105]. It is a highly aggressive malignancy with reported overall 5-year survival ranging from 7–20% [103,106], declining substantially with advancing stage. The global incidence and mortality of CCA vary considerably, with the highest rates reported in Southeast Asia (85-1.2 cases per 100,000) largely due to the high prevalence of liver fluke infections, namely *Opisthorchis viverrini* (*O. viverrini*) [107,108,109], compared to 3.4-0.3 cases per 100,000 in Western countries [110,111]. Anatomically, CCA is classified into intrahepatic (iCCA) and extrahepatic (eCCA) subtypes [110].

Diagnosis currently relies on a combination of biochemical tests, imaging modalities and, ultimately, histopathological confirmation. However, because early-stage disease is frequently asymptomatic, approximately ~70% of patients are diagnosed at an advanced stage [110,112]. Imaging techniques, including ultrasound, computed tomography (CT), magnetic resonance imaging (MRI) and magnetic resonance cholangiopancreatography (MRCP), play a central role in diagnosis but have limited accuracy, particularly for early disease. The serum biomarkers carbohydrate antigen 19-9 (CA19-9), cancer antigen 125 (CA-125) and carcinoembryonic antigen (CEA) are commonly used to support diagnosis, although none possess sufficient SN or SP to serve as a standalone diagnostic tool, and tissue biopsy remains necessary for definitive confirmation [109]. Consequently, there is a pressing need for improved non-invasive diagnostic approaches.

Although the literature on urinary biomarkers for CCA remains limited, the available studies suggest that urine-based analyses may have value for both diagnosis and risk stratification (Table 6). Urinary metabolic profiling has demonstrated the ability to distinguish iCCA from controls and to enhance diagnostic performance when combined with CA19.9 [113]. In regions endemic for *O. viverrini*, urinary biomarkers reflecting oxidative stress or parasitic antigen burden have also shown the potential for identifying individuals at increased risk of CCA and for monitoring treatment response [114]. The key limitations of these studies include small cohort sizes, limited subtype stratification, and absence of longitudinal or early-disease studies to assess clinical relevance.

### 3.6. Pancreatic Cancer

Pancreatic ductal adenocarcinoma (PDAC) is among the most lethal malignancies, with approximately 510,500 new cases and 467,000 deaths worldwide in 2022 [1]. In the UK, around 10,800 new cases are diagnosed annually, and PDAC accounts for approximately 6% of all cancer deaths [116]. Five-year survival remains poor, at around 10% in the UK and 12% in the US [116,117], largely because the disease is typically asymptomatic at early stages. More than 80% of patients present with locally advanced or metastatic disease, and almost half are diagnosed following emergency presentation. Since surgical resection remains the only potentially curative treatment, early diagnosis is critical; patients with small, localised tumours can achieve 5-year survival rates exceeding 60% [118,119].

Currently, there are no validated biomarkers for the early detection of PDAC in clinical use. The only clinically approved biomarker, CA19-9, is used primarily for disease monitoring because of its insufficient SN and SP for diagnosis [120,121]. Since our previous review of liquid biopsy biomarkers for PDAC [122], relatively limited progress has been made in the development of urinary biomarkers (Table 7).

The most advanced urinary biomarker test is a three-protein panel comprising REG1B, LYVE1 and TFF1, originally identified by Radon et al. [123] and subsequently validated in 590 urine samples [124]. Combined into the PancRISK algorithm, the panel stratifies individuals into average- and elevated-risk groups [125]. Importantly, when combined with serum CA19-9, these biomarkers were capable of identifying PDAC up to two years before clinical diagnosis [126]. Several subsequent studies have applied machine-learning approaches to the same dataset, further supporting the robustness of this biomarker panel [127,128,129,130]. Among urinary miRNA studies, the most comprehensive was reported by Baba et al. [131]. Using RNA sequencing, the authors identified a signature comprising 26 upregulated and 19 downregulated miRNAs and validated it in two independent cohorts enriched for stage I/IIA disease. The biomarker panel achieved an AUC of 0.963, with 77.8% SN and 95.7% SP. VOCs have also attracted interest. Six studies used the N-NOSE platform, which exploits the odour-sensing capability of *Caenorhabditis elegans*. In the largest study, Kobayashi et al. [132] distinguished 104 PDAC cases from 95 controls with 84.6% SN and 60.0% SP. Notably, the assay detected stage 0–I PDAC and correlated more strongly with early-stage than advanced disease. Collectively, these findings suggest that urinary protein and miRNA biomarkers are the most advanced candidates for PDAC early detection, although large prospective validation studies remain necessary before clinical implementation. 

**Table 7:** Urinary biomarkers in pancreatic ductal adenocarcinoma (PDAC).

Biomarker Type	Biomarker(s)	Study Population	Method	AUC (95% CI)	SN% (95% CI)	SP% (95% CI)	Reference
Proteins	REG1B + LYVE1 + TFF1 (panel) ±CA19-9	199 PDAC (102 Stage I/II), 208 BeC, 183 H	ELISA, LR	PDAC I/II v H: panel, Training 0.93 (0.90–0.96); Validation 0.93 (0.88–0.98); panel + CA19-9, 0.99 (0.98–1). PDAC I/II v BeC: panel 0.86 (0.81–0.91); panel +CA19-9, 0.91 (0.88–0.95)	PDAC I/II v H: panel, Training and Validation > 85; panel + CA19-9, 96 (0.91–1). PDAC I/II v BeC: panel 88 (0.81–0.95); panel + CA19-9, 80 (0.71–0.88)	PDAC I/II v H: panel, Training & Validation > 85; panel +CA19-9, 96 (0.92–1). PDAC I/II v BeC: 76 (0.67–0.84); panel +CA19-9, 89.8 (0.83–0.95)	[124]
Proteins	REG1B + LYVE1 + TFF1 (panel) ±CA19-9	99 Prediagnostic PDAC (0 to 5 years before diagnosis), 198 H	ELISA, LR	PDAC v H, panel + CA19-9: Training 0–1 year 0.89 (0.82–0.96), 0–2 years 0.77 (0.69–0.85); Validation 0–1 year 0.82 (0.72–0.92), 0–2 years 0.72 (0.63–0.81)	PDAC v H, panel + CA19-9: at fixed SP 75, 0–1 year 83, 0–2 years 65; at fixed SP 80: 0–1 year 77, 0–2 years SN 60	PDAC v H, panel + CA19-9: at fixed SN 75, 0–1 year 85, 0–2 years 57; at fixed SN 80: panel + CA19-9, 0–1 year 77, 0–2 years SP 53	[126]
Proteins	CRP	176 PDAC, 265 BeC, 93 H	ELISA, LR	PDAC v H 0.67 (0.61–0.74); PDAC v BeC 0.66 (0.61–0.72); PDAC v H + BeC 0.65 (0.60–0.71)	At CRP cut-off 6 ng/mL: 30	At CRP cut-off 6 ng/mL: PDAC v H 99; PDAC v H + BeC 94	[133]
Proteins	CD276 fragment (peptide #13684)	Exploratory: 39 PDAC, 42 H Validation: 28 PDAC, 36 H	iTRAQ- MALDI-TOF MS	PDAC v H, Validation: CD276, 0.86; CA19-9, 0.85; CD276 + CA19-9, 0.88	PDAC v H, Validation: CD276, 75; CA19-9, 66; CD276 + CA19-9, 93	PDAC v H, Validation: CD276, 89; CA19-9, 91; CD276 + CA19-9, 83	[134]
miRNAs	MIR1246	41 PDAC, 30 H	qRT-PCR	PDAC v H 0.90	PDAC v H 90.2	PDAC v H 83.3	[135]
miRNAs	miR-3940-5p/miR-8069 ratio	Discovery: 9 PDAC (2 Stage I/II), 7 H Validation: 43 PDAC (15 Stage I/II), 12 CP, 25 H	miRNA expression profiling + 3D digital PCR	PDAC v CP+ H 0.73 (0.62–0.84)	PDAC v CP+ H 58.1 (at AUC 0.94); miR-3940-5p/miR-8069 ratio + CA19-9, 93	PDAC v CP+ H 89.2 (at AUC 0.94); miR-3940-5p/miR-8069 ratio + CA19-9, 70.3	[136]
miRNAs	miR-210-3p	Validation: 58 PDAC (41 IDC, 17 IPMC), 41 H	Toray^®^ 3D-Gene; qRT-PCR	PDAC v H 0.79; IPMC v H 0.76	PDAC v H 82.8; IPMC v H 94	PDAC v H 74.3; IPMC v H 63	[137]
Exosomal miRNAs	45 microRNAs	Training: 99 PDAC (33 Stage I/IIA), 216 BeC Validation: 54 PDAC (9 Stage I/IIA), 93 BeC	small RNA-seq	PDAC v BeC, Training 0.97 (0.92–0.99); Validation 0.96 (0.93–0.98)	PDAC v BeC, Training 93.9 (87.5–97.3); Validation 77.8 (64.9–87.3); PDAC I/IIA v BeC, Training 97 (83.9–99.8); Validation 77.8 (44.2–95.9)	PDAC v BeC, Training 91.7 (87.1–94.7); Validation 95.7 (89.4–98.5)	[131]
VOCs	VOC profile (52,200 measuring points per sample)	68 PDAC, 36 AP, 18 CP, 7 PLP, 53 H	FAIMS	NR	PDAC v H 79 (0.68–0.87); PDAC + PLP v H 85 (0.74–0.91); PDAC + PLP + AP + CP v H 91 (0.84–0.95)	PDAC v H 79 (0.66–0.88); PDAC + PLP v H 75 (0.72–0.85); PDAC + PLP + AP + CP v H 47 (0.33–0.61)	[138]
VOCs	Polyamine signature, panel 1 (four of 14): acetylputrescine + diacetylspermidine + N8-acetylspermidine + diacetylputrescine	68 PDAC, 36 AP, 18 CP, 7 PLP, 53 H	MS; quadratic discriminant; leave-one-out cross-validation	PDAC + PLP v H 0.88	PDAC + PLP v H 94	PDAC + PLP v H 68	[139]
VOCs	2,6-dimethyl-octane, nonanal, 4-ethyl-1,2-dimethyl-benzene and 2-pentanone	45 PDAC, 45 CP, 33 H	GC-IMS, GC-TOF-MS; GP, SLR	GC-IMS: PDAC v all 0.69 (0.58–0.79); PDAC v H 0.88 (0.79–0.97); PDAC v CP 0.58 (0.45–0.71) GC-TOF-MS: PDAC v all 0.75 (0.63–0.87); PDAC v H 0.86 (0.75–0.97); PDAC v CP 0.55 (0.37–0.73)	GC-IMS: PDAC v all 72 (0.56–0.85); PDAC v H 84 (0.69–0.73); PDAC v CP 51 (0.35–0.67) GC-TOF-MS: PDAC v all 52 (0.37–0.67); PDAC v H 72 (0.51–88); PDAC v CP 38 (0.18–0.62)	GC-IMS: PDAC v all 60 (0.57–0.71); PDAC v H 94 (0.79–0.99); PDAC v CP 73 (0.56–0.85) GC-TOF-MS: PDAC v all 96 (0.79–1); PDAC v H 96 (0.79–1); PDAC v CP 88 (0.68–0.97)	[140]
*C. elegans* VOCs	VOCs	24 PDAC (14 Stage I/II), 76 H	N-NOSE	PDAC v H 0.86; PDAC I/II v H 0.85	NR	NR	[76]
*C. elegans* VOCs	VOCs	Discovery: 83 PDAC; Validation: 11 PDAC (Stages 0/IA), 17 H	N-NOSE	Before vs. after surgical removal: (10× urine dilution) 0.84, (100× dilution) 0.82	NR	NR	[141]
*C. elegans* VOCs	VOCs	104 PDAC, 95 BeC	N-NOSE	PDAC v BeC, accuracy 72.86	PDAC 0/IV, 84.6; PDAC 0, 100; I, 87.5; II, 93.33; III, 84.21; IV, 80.95	PDAC v BeC 60	[132]
*C. elegans* VOCs	VOCs	46 PDAC, 11 CP, 31 BeC	SIFT-MS	0.98 (0.95–1.00)	PDAC v CP 93.5	PDAC v CP 100	[142]
*C. elegans* VOCs	VOCs	32 cancer patients (only 2 PDAC), 143 H	N-NOSE	All cancers v H, 10× urine dilution 0.92; 100× dilution 0.90	All cancer v H, 10× urine dilution 78.1; 100× dilution 75.1 (PDAC 50)	All cancer v H, 10× urine dilution 93.7; 100× dilution 95.1	[41]
*C. elegans* VOCs	VOCs	63 PDAC (among 1664 patients with over 20 different cancer types)	N-NOSE	NR	PDAC 0/IV 77.8; PDAC I, 75; II, 77.3; III, 100; IV, 74.2	NR	[40]
Metabolites	Panel of 6: trigonelline, glycolate, hippurate, creatine, myoinositol, hydroxyacetone	Discovery: 56 PDAC; Validation: 36 PDAC, 56 H	1 H NMR	PDAC v H, Discovery: 0.93 (0.87–0.98), Validation: 0.86 (0.75–0.96); PDAC I/II v H, 0.89 (0.81–0.95)	PDAC v H, Discovery: 89.3, Validation: 80.6; PDAC I/II v H 83	PDAC v H, Discovery: 83.9, Validation: 85.7; PDAC I/II v H 80.4	[143]
Metabolites	Panel of 4 free-glycans (#2, #7, #8, #11)	55 PDAC, 120 H	Chromatography	PDAC v H 0.91	NR	NR	[144]
Metal elements	Ca, Mg, Cu, Zn	21 PDAC, 46 H	Q-ICP-MS	PDAC v H: Mg, 0.78 (0.66–0.90); Ca, 0.79 (0.66–0.92); Zn, 0.68 (0.51–0.85); Cu, 0.67 (0.48–0.85); Mg + Ca + Zn + Cu, 0.99 (0.97–1)	PDAC v H: Mg, 61 (0.38–0.81); Ca, 76 (0.57–0.90); Zn, 57 (0.38–0.76); Cu, 66 (0.47–0.85); Mg + Ca + Zn + Cu, 95 (0.85–1)	PDAC v H: Mg, 89 (0.80–0.97); Ca, 82 (0.71–0.93); Zn, 84 (0.74–0.93); Cu, 85 (0.74–0.93); Mg + Ca + Zn + Cu, 97 (0.93–1)	[17]

AP, acute pancreatitis; CP, chronic pancreatitis; CRP, C-reactive protein; H, healthy; IDC, invasive ductal carcinoma; IPMC, intraductal papillary mucinous carcinoma; LR, logistic regression; NR, not recorded; PLP, premalignant lesion of pancreas.

### 3.7. Colorectal Cancer

Colorectal cancer (CRC) is the second leading cause of cancer-related mortality worldwide, with more than 1.9 million new cases and over 930,000 deaths reported in 2020. The World Health Organization (WHO) estimates that the global burden will continue to rise, reaching approximately 3.2 million new cases and 1.6 million deaths annually by 2040 [145]. Although the relative 5-year survival rate across all stages is around 65.4%, it is significantly higher (91.3%) for patients diagnosed early [146]. Current screening programmes based on faecal immunochemical testing (FIT) and faecal occult blood testing (FOBT) are effective but suffer from suboptimal uptake and compliance [147,148]. The development of urinary biomarkers could potentially provide a more acceptable alternative to stool-based tests and therefore improve participation in population screening programmes.

Recent studies have highlighted the potential of urinary biomarkers as non-invasive tools for CRC detection, encompassing metabolites, proteins and nucleic acids (Table 8). Among these, metabolomic profiling has shown particularly encouraging results. Panels incorporating metabolites such as diacetylspermine and kynurenine achieved good diagnostic performance (AUC 0.864; SN and SP approximately 80%), while urinary D-amino acid profiles demonstrated exceptionally high accuracy (AUC 0.998; SN 98.3%; SP 96.8%) [149,150,151]. Additional metabolomic studies employing nuclear magnetic resonance (NMR) spectroscopy and liquid chromatography-tandem mass spectrometry (LC-MS/MS) identified markers including taurine, hippurate and glutamate, reporting AUCs ranging from 0.823 to 0.877 and SN of up to 96% [151,152]. Urinary protein biomarkers have also demonstrated promising diagnostic potential. Dipeptidase 1 (uDPEP1) and trefoil factor 1 (uTFF1) were among the most promising candidates, particularly for early-stage disease detection, achieving AUC values of up to 0.956 and SN of up to 93.8% [153]. Similarly, urinary nucleic acid biomarkers have shown encouraging performance. DNA methylation markers, including SEPT9 and SDC2, together with RNA-based markers such as miR-129-1-3p and miR-566, achieved AUCs ranging from 0.78 to 0.868, with reported SN between 70% and 99.9% [154,155]. VOC-based approaches represent another rapidly developing area. Urinary VOC signatures analysed using gas chromatography-mass spectrometry (GC-MS) or ion mobility spectrometry demonstrated high diagnostic accuracy, with AUCs reaching 0.98 [156,157,158]. However, distinguishing advanced adenomas from healthy controls remains challenging. Emerging technologies have further expanded the field. Surface-enhanced Raman spectroscopy (SERS) and modular detection platforms targeting matrix metalloproteinases (MMPs) achieved excellent diagnostic performance, with AUCs ranging from 0.959 to 0.977 and SN and SP all above 80% [159,160].

Collectively, these findings demonstrate the considerable promise of urinary biomarkers for CRC detection, particularly when incorporated into multi-marker panels. While several approaches already show excellent diagnostic performance, most studies remain limited by cohort size and a lack of independent validation. 

### 3.8. Renal Cell Carcinoma

Kidney cancer is the 14th most common cancer worldwide, with 434,419 new cases and 155,702 deaths reported in 2022 [1]. In the UK, approximately 13,900 new cases are diagnosed annually, representing a 47% increase over the past decade [165]. Renal cell carcinoma (RCC) accounts for around 90% of adult kidney cancers, with rising incidence partly attributable to increased incidental detection through imaging [166]. Despite this, 20–30% of patients present with advanced or metastatic disease, where 5-year survival falls from 70–90% in localised disease to below 10% [167,168]. Early RCC is typically asymptomatic, and fewer than 30% of cases are diagnosed based on symptoms alone [169]. Current diagnosis relies on imaging, supported by blood tests and increasingly by renal mass biopsy, although false-negative results remain a limitation. There are currently no clinically approved biomarkers for early RCC detection [167,168,169]. Given its direct contact with the renal epithelium, urine represents an ideal non-invasive source of tumour-derived biomarkers. Our search identified 41 studies evaluating urinary biomarkers for RCC, which could be broadly grouped into microRNAs, metabolites, proteins, and DNA methylation markers (Table 9).

Small non-coding RNAs were investigated in 11 studies; of these seven were microRNAs [170,171,172,173,174,175,176,177,178,179,180], although no clear diagnostic candidate emerged. Most studies were small, with five including fewer than 30 RCC cases. One notable finding was urinary exosomal miR-30c-5p, which showed differential expression in clear-cell RCC (ccRCC) compared with healthy controls (AUC 0.82, SN 68.6%, SP 100%) and was linked to tumour inhibition through targeting heat-shock protein 5 (HSP5) [177]. Metabolomic approaches appeared the most consistently promising [181,182,183,184,185,186,187,188,189,190,191,192]. Liu et al. [186] identified a nine-metabolite urinary signature using LC-MS that distinguished RCC from controls with an AUC of 0.808 (84.8% SN, 83.8% SP). These findings were supported by subsequent studies [188,189,192]. Combining LC-MS and NMR, Bifarin et al. [184] reported a seven-metabolite panel with an AUC of 0.98, SN of 94%, and SP of 85%. Urinary VOCs were evaluated in four studies [193,194,195,196]. The largest study by Holbrook et al. [194], included 233 RCC cases and identified a 24-VOC model with excellent diagnostic performance (AUC 0.94, 86% SN, 92% SP). Nine studies assessed urinary proteins [197,198,199,200,201,202,203,204,205]. Phosphoproteomic analysis of extracellular vesicles demonstrated encouraging performance (AUC 0.96 and 0.92) in small cohorts [199]. However, previously proposed markers, including AQP1 and PLIN2 [206,207,208], showed inconsistent results in later validation studies [197,202]. A recent review highlighted substantial methodological heterogeneity and limited evidence for their clinical utility [209]. Finally, four studies evaluated urinary DNA methylation markers [210,211,212,213], but small cohorts and limited validation preclude firm conclusions. Overall, urinary metabolomic and VOC-based approaches currently appear the most promising for RCC early detection, although large prospective validation studies are required before clinical implementation.

**Table 9:** Urinary biomarkers in renal cancer (RCC).

Biomarker Type	Biomarker(s)	Study Population	Method	AUC (95% CI)	SN (95% CI)	SP (95% CI)	Reference
miRNAs	Panel miR-122, miR-1271, miR-15b	13 RCC, 14 H	RT-qPCR	0.96 (0.88–1.04)	100 (75–100)	86 (57–98)	[172]
miRNAs	Panel miR-122, miR-1271, miR-15b (validation of [172])	28 ccRCC, 28 H	RT-qPCR and 7-p urinary score	0.81	96	65	[171]
miRNAs	MIR200a	27 ccRCC, 27 H	RT-qPCR	0.82 (0.77–0.93)	NR	NR	[178]
miRNAs	miR1275	18 RCC, 16 H	NGS (HTG EdgeSeq miR WTA)	0.95	94.12	93.75	[170]
miRNAs	Panel miR-200a-3p + miR-34a-5p + miR-365a	Training: 16 RCC, 16 H; Validation: 51 RCC, 32 H	qRT-PCR	Training: 0.81 (0.64–0.97); Validation: 0.65 (0.51–0.79)	NR	NR	[174]
EVs. miRNAs	miR-30c-5p	70 RCC, 30 H	NGS-RTqPCR	0.82 (0.73–0.89)	68.57	100	[177]
EVs. miRNAs	miR-135b-5p, miR-200c-3p, and miR-203a-3p	125 RCC (114 Stage I), 128 H	qRT-PCR	0.78 (0.72–0.84); RCC Stage I v H, 0.78 (0.72–0.84)	NR	NR	[180]
sncRNAs	circEGLN3, circSOD2	76 RCC, 8 BeC, 19 H	qRT-PCR	RCC v all, circEGLN3 0.71, circSOD2 0.68	circEGLN3 69; circSOD2 100	NR	[175]
snoRNAs	SNORD15A, SNORD35B, SNORD60 (from TCGA and SNORic)	100 RCC, 131 H	qRT-PCR	0.74, 0.71, 0.68, respectively; Combined: RCC early stages vs H 0.74	61.8, 61.8, 64.9, respectively; Combined: RCC early stages vs H 89	79, 74, 68, respectively; Combined: RCC early Stages v H 57.3	[179]
snoRNAs	SNORD63, SNORD96A	75 RCC, 96 H	qRT-PCR	0.70, 0.67; SNORD96A RCC early stages vs H 0.93	47.9, 53.1; SNORD96A RCC early stages vs H 95	86.7, 77.3; SNORD96A RCC early Stages v H 87	[176]
EVs. snoRNAs	SNORD99, SNORD22, SNORD26, SNORA50C	Discovery: 47 ccRCC, 16 BeC Validation: 54 ccRCC, 24 BeC	NGS; qRT-PCR	0.68 (0.58–0.78), 0.70 (0.60–0.80), 0.68 (0.57–0.78), 0.73 (0.64–0.83) respectively; SNORAS0e + SNORD99: 0.77 (0.68–0.87)	66.7, 50, 76.1, 77.8, respectively; SNORAS0e +SNORD99: 90	62.5, 87.5, 66.7, 66.7, respectively; SNORAS0e +SNORD99: 62.5	[173]
Lipid metabolites	DSGb5 (GSL)	15 RCC, 9 H	LC-MS	RCC v H 0.88 (0.75–1.01)	RCC v H 85.7	RCC v H 88.9	[214]
Lipid metabolites	lactosylsulfatides; sulfatides with hydroxylated fatty acyls	76 RCC, 67 H	MALDI LTQ Orbitrap XL-MS	0.86 (0.76–0.95)	74.2 (55.4–88.1)	88.9 (70.8–97.7)	[185]
Metabolites	α-CEHC, β-cortolone, deoxyinosine, flunisolide, 11b,17a,21-trihydroxypreg-nenolone, glycerol tripropanoate	21 RCC w hematuria, 94 RCC w/o hematuria; 69 BCa w hematuria; 77 BCa w/o hematuria; 142 H	UPLC-MS	RCC v H: 0.95	NR	NR	[190]
Metabolites	Panel 1: androstenedione,7-alpha-hydroxy-3-oxochol-4-en-24-oic acid, lithocholyltaurin, 11-dodecenoic acid,tetrahydroaldosterone-3-glucuronide, cortolone-3-glucuronide. Panel 2: folate metabolism, tryptophan metabolism, biopterin metabolism; N-formylkynurenine	100 RCC, 34 Be kidney tumour, 129 H	LC-MS	RCC v H: Panel 1 + N-Jasmonoyltyrosine + dopamine 4-sulfate + methylazelaic acid, 0.88; RCC vs. Be kidney tumor: L-3-hydroxykynurenine + 1,7-dimethylguanosine + tetrahydroaldosterone-3-glucuronide, 0.81; RCC v All controls: 0.80	RCC v H: Panel 1 + N-Jasmonoyltyrosine + dopamine 4-sulfate + methylazelaic acid, 85.1; RCC v Be kidney tumor: L-3-hydroxykynurenine + 1,7-dimethylguanosine + tetrahydroaldosterone-3-glucuronide, 74.6; RCC v All controls: 84	RCC v H: Panel 1 + N-Jasmonoyltyrosine + dopamine 4-sulfate + methylazelaic acid, 87.5; RCC v Be kidney tumor: L-3-hydroxykynurenine + 1,7-dimethylguanosine + tetrahydroaldosterone-3-glucuronide 80.0; RCC v All controls, 83.8	[186]
Metabolites	Lactate, 2-hydroxyglutarate, L-glutamate, myoinositol, d-sedoheptulose 7-phosphate	87 ccRCC, 60 BeC	LC-MS	lactate + 2-hydroxyglutarate + L-glutamate + myoinositol + d-sedoheptulose 7-phosphate, 0.96; RCC early stages v H, ctrls:p-cresol + octanal + benzaldehyde + 3- methylbutanal + 4-heptanone + 2-furaldehyde, 0.79	lactate + 2-hydroxyglutarate+ L-glutamate+ myoinositol+ d-sedoheptulose 7-phosphate, 93.1; p-cresol + octanal + benzaldehyde + 3- methylbutanal + 4-heptanone + 2-furaldehyde, 84	lactate + 2-hydroxyglutarate + L-glutamate + myoinositol + d-sedoheptulose 7-phosphate, 95; p-cresol + octanal + benzaldehyd+ 3- methylbutanal + 4-heptanone + 2-furaldehyde, 73	[189]
Metabolites	p-cresol glucuronide	50 ccRCC, 50 H	UPLC-QTOF-MS untargeted metabolomics	0.66 (0.56–0.76)	57 (44.8–68.2)	72 (57.5–82.7)	[188]
Metabolites	Panel 1: cortolone, testosterone and L-2-aminoadipate adenylate. Panel 2: aminoadipic acid, 2-(formamido)-N1-(5-phospho-d-ribosyl) acetamidine, alpha-N-phenylacetyl-l-glutamine	Discovery: 39 RCC, 22 Be renal tumors, 68 H	LC-MS	Panel 1: RCC v Be renal tumors, ten-fold cross-validation 0.86, Validation 0.87. Panel 2: RCC v H, ten-fold cross-validation 0.84, Validation 0.89	NR	NR	[192]
Metabolites	Seven-metabolite panel	105 RCC, 179 BeC	LC-MS, NMR, ML	0.98	94	85	[184]
Metabolites	Glycine, citrate	41 Early Stage RCC, 29 Late Stage RCC, 174 H	LC-MS, Non-magnetic resonance, ML	Combination of 24 metabolites: 4-guanidinobutanoic acid + 7-aminomethyl-7-carbaguanine + N-alpha-dimethyl-L-histidine + diethyl-2-methyl-3-oxosuccinate + 3-hydroxyanthranilic acid + ADP-forming acetyl-CoA synthetase + lys-gly/gly-lys + succinic anhydride + citrate + glycine + choline + acetone + pyruvate + cytosine dimer + dihydrouridine + hydroxyprolyl-asparagine, 0.96	Combination of 24 metabolites: 80	Combination of 24 metabolites: 93	[183]
Metabolites	15 metabolites: serine, heptanol, 3-methylene-indolenine, 2-methyl-3-hydroxy-5-formylpyridine-4-carboxylate, phosphodimethylethanolamine, 4-methoxyphenylacetic acid, N-acetylglutamine, 3,5-dihydroxyphenylvaleric acid, hydroxyhexanoylglycine, valyl-leucine, leucyl-histidine, oleamide, 9,12,13-trihydroxyoctadecenoic acid, stearidonyl carnitine and squalene	50 ccRCC, 50 H	AuNPET, SALDI MS	(In order): 0.561, 0.757, 0.678, 0.716, 0.579, 0.600, 0.813, 0.844, 0.628, 0.706, 0.661, 0.599, 0.741, 0.658, 0.634. Combination of all 15 biomarkers: 0.91	NR	NR	[181]
Metabolites	9,12,13-Trihydroxyoctadecenoic acid; 3-Hydroxydecanoyl carnitine	50 ccRCC, 50 H	AuNPET, SALDI MS	0.66, 0.66, respectively	NR	NR	[182]
Metabolites	Group 1: myo-Inositol, Creatine, Sucrose, Trigonelline, 2-Furoylglycine, Urea, 4-Hydroxyphenylacetate, Alanine, Homovanillate, Glycolate, N-Dimethylglycine, Isoleucine. Group 2: Succinylacetoacetate, Cys-Gly-Ser-His, His-Gly-Ser-Ser, Met-Thr-His	50 ccRCC, 50 H	Group 1: 1H NMR spectral analyses; group 2: AuNPET, SALDI MS	Group 1 (in order): 0.96, 0.91, 0.86, 0.86, 0.85, 0.83, 0.82, 0.81, 0.81, 0.8, 0.8, 0.76. Group 2 (in order): 0.76, 0.77, 0.77, 0.77	NR	NR	[187]
Metabolites	Metabolic biomarkers (4 specific for ccRCC)	221 ccRCC (181 Stage I/II), 95 pRCC, 99 chRCC, 41 Be renal tumours, 200 H	NELDI MS	All RCC v H 0.93 (0.88–0.96); all RCC v Be tumors 0.85 (0.82–0.91); ccRCC 0.92 (0.88–0.94)	All RCC v H 90.8; all RCC v Be tumors 83.9; ccRCC Stage I/II 90.5	All RCC v H 89.9; all RCC v Be tumors 84.6; ccRCC Stage I/II 91.3	[191]
Metabolites-VOC	p-cresol, octanal, benzaldehyde; 4-heptanone, 2-furaldehyde, 3-methylbutanal	75 ccRCC, 75 H	HS-SPME-GC-MS	(in order): 0.71, 0.61, 0.62, 0.70, 0.61, 0.80, 0.76; p-cresol + octanal + benzaldehyde + 3- methylbutanol, + 4-heptanone + 2-furaldehyde: 0.87	p-cresol + octanal + benzaldehyde + 3- methylbutanol, + 4-heptanone + 2-furaldehyde, 0.83	p-cresol + octanal + benzaldehyde + 3- methylbutanol, + 4-heptanone + 2-furaldehyde, 0.79	[196]
VOCs	nanocomposite array of 32 organic polymer sensors	110 RCC, 142 H	Electric nose (Cyranose 320, Smith Detections, Pasadena, CA)	>0.85	71.8	88.7	[193]
VOCs	24 VOC markers	Training: 163 ccRCC, 31 H Validation: 70 ccRCC, 12 H	SBSE-TD-GC/MS; LR	Training: 0.98 (0.93–1); Validation: 0.94 (0.87–1)	Training 99; Validation 86	Training 97; Validation 92	[194]
VOCs	8-VOC panel (including cyclohexanone, 1-hexanol-2-ethyl up in RCC)	40 ccRCC, 8 H	GC/MS and Gas Sensor	Discovery: Training 0.98 (0.94–1.02), Test 0.88 (0.77–0.98); Validation: Training 0.98 (0.94–1.02), Test 0.91 (0.82–1.00)	Discovery: Training 85.7, Test 83.3 Validation: Training 100, Test 100	NR	[195]
Proteins	aquaporin-1 (uAQP-1)	34 RCC, (2 other subtypes), 24 H	ELISA	0.52 (0.42–0.62)	47.2	66.7	[197]
Proteins	aquaporin-1 (uAQP-1)	69 ccRCC, 53 H	ELISA	NR	48.5	67.3	[202]
Proteins	aminin-5, collagen IV, fibronectin	60 ccRCC, 26 BeC, 54 H	SPRi biosensors	fibronectine 0.47; collagene IV 0.94; laminin 0.92	fibronectine 20; collagene IV 78; laminin 100	fibronectine 100; collagene IV 100; laminin 92	[198]
Proteins	PKHD1L1, ANGPTL6, FABP4 and C3 of 133 found	Discovery: 12 ccRCC, 11 BeC Validation: 46 ccRCC, 45 BeC	LC-MS/MS; ELISA	0.83	NR	NR	[205]
Proteins	APOC3, UMOD and CILP of 18 validated proteins	Discovery (DIA cohort): 89 ccRCC, 90 H Validation (RPM cohort): 58 ccRCC, 36 H	LC-MS/MS and ELISA	DIA cohort: 0.88; PRM cohort: 0.93	DIA cohort: 0.88; PRM cohort: 0.9	DIA cohort: 0.76; PRM cohort: 0.93	[204]
Proteins	peptides	40 ccRCC, 15 Be kidney neoplasm, 30 H	MALDI-TOF MS; GA, SNN, QC algorithms	(cross-validation) RCC v H, GA 97.5; RCC v BeC, GA 85.9	RCC v H, GA 100; RCC v BeC, GA 96; Validation: 88.7	RCC v H, GA 95; RCC v BeC, GA 75.9; Validation: 73.2	[200]
Proteins	adhesion molecules and defense proteins: three protein-panel CEPT + SEZ6L2 + COX5B	27 ccRCC, 27 H	LC-MS	0.97	92.6	92.6	[201]
Cancer-retina antigens	arrestin, recoverin, rhodopsin kinase, and transducin	89 ccRCC, 50 H	multiplex immunoassay	arrestin+ recoverin: 0.96 (0.93–0.99)	arrestin+ recoverin: 96	arrestin+ recoverin: 92	[203]
EV Phospho-proteomics	EV Phospho-proteomic profile	28 ccRCC (14 low grade, 14 high grade), 15 H	LC-MS/MS	5-phosphosite signature: RCC low grade v H, 0.96; RCC high grade v H, 0.92. 7-phosphosite signature: RCC low grade v H, 0.96; RCC high grade v H, 0.92	NR	NR	[199]
cfDNA methylation	Top 300 DMRs	30 RCC (TNM Stage: 17 I, 3 II, 4 III, 6 IV), 15 H	cfMeDIP-seq, NGS	0.85 (0.83–0.88)	NR	NR	[211]
cfDNA methylation (Urine sediment)	miR-30a-5p promoter methylation	Training: 53 ccRCC (Stage: 26 I, 4 II, 18 III, 5 IV), 57 H Validation: 171 ccRCC (Stage: 121 I, 8 II, 31 III,11 IV), 85 H	qMSP	Training: 0.68 Validation: 0.67	Training: 83 Validation: 63	Training: 53 Validation: 67	[212]
DNA methylation	TAC1, PCDH8, ZNF677,FLRT2	39 ccRCC, 92 H	qMSP assay	Panel: 0.74	Panel: 92	Panel: 52	[213]
DNA methylation	ZNF677, FBN2, PCDH8, TFAP2B, TAC1, FLRT2	217 ccRCC, 92 H	qMSP assay	ZNF677 + PCDH8 (w or w/o FBN2 or FLRT2): 0.78	ZNF677 + PCDH8 (w or w/o FBN2 or FLRT2): 69–78	ZNF677 + PCDH8 (w or w/o FBN2 or FLRT2): 69–80	[210]

BCa, bladder cancer; ccRCC, clear cell RCC; chRCC, chromophobe RCC; DMR, differentially methylated regions; EVs, extracellular vesicles; GA, genetic algorithm; GSL, Gly-cosphingolipids; H, healthy; LR, logistic regression; NR, not recorded; pRCC, papillary RCC; sncRNA, small non-coding RNA; QC, quick classifier; SNN, supervised neural network; snoRNA, small nucleolar RNA.

### 3.9. Ovarian Cancer

Ovarian Cancer (OC) is the leading gynaecological malignancy in the UK and accounts for more deaths than all other gynaecological malignancies combined. Approximately two thirds of women in the UK are diagnosed when the disease is at an advanced stage and approximately 27% present via emergency services [215]. Worldwide, OC remains the gynaecological cancer with the highest mortality [37]. The poor prognosis is largely attributable to late diagnosis, as early symptoms are often vague and non-specific. Currently, CA125 is the most widely used biomarker to support diagnosis; however, it is elevated in only around 50% of early-stage cases and lacks SP, being increased in several other malignancies and benign conditions. Its limited SN and SP, particularly for early detection, remain major clinical challenges [6,216,217,218].

Our search identified a range of urinary biomarkers with potential diagnostic utility for OC (Table 10). Interestingly, although CA125 is most commonly assessed in blood, several studies have also explored its measurement in urine. Of the 10 ovarian cancer studies included in this review, six focused on urinary protein biomarkers, three of which evaluated urinary CA125. When assessed alone, urinary CA125 demonstrated limited diagnostic performance [219]. However, when combined with other biomarkers, particularly urinary HE4 and osteopontin (OP), its performance improved and was broadly comparable to serum CA125, although these studies were limited by relatively small cohorts and, in some cases, wide confidence intervals [220,221]. Notably, a plasmonic gold chip assay incorporating urinary CA125, HE4 and OP achieved SN and SP of 90.2% and 80.3%, respectively [220].

Investigations concerning metabolomic profiling were also among the results and generally demonstrated favourable diagnostic performance, with good SN and SP estimates. L-pyroglutamic acid, turanose, glycine, L-threonine, and L-tyrosine were identified by Eroglu et al. as important in distinguishing tumour from benign disease, while ten metabolite panels were found to have an AUC of 0.887 for distinguishing cancer from benign disease. Importantly, these studies often included both healthy and benign disease controls, strengthening the clinical relevance of their findings. However, cohort sizes remained relatively small, limiting confidence in the general application of the results [222,223].

Although no urinary biomarker has yet reached clinical practice for the detection of ovarian cancer, the breadth of research identified in this review provides a strong foundation for future development, but larger prospective studies and independent validation cohorts are required before these approaches can be translated into clinically applicable diagnostic tests.

### 3.10. Ampullary and Duodenal Cancers

The search for urinary biomarkers of ampullary cancer identified only one publication each in PubMed and Web of Science, and 19 publications in Embase. Following detailed screening, none of these met the predefined inclusion criteria. Similarly, no studies relevant to urinary biomarkers for duodenal cancer fulfilled the inclusion criteria, despite the large number of records retrieved during the initial search (148 from PubMed, 1265 from Embase, and 12 from Web of Science). This absence of eligible studies likely reflects the rarity of these malignancies and consequently, the limited research efforts devoted to developing non-invasive diagnostic approaches for their early detection utilising urine specimens.

**Table 10:** Urinary biomarkers in ovarian cancer (OC).

Biomarker Type	Biomarker(s)	Study Population	Method	AUC (95% CI)	SN (95% CI)	SP (95% CI)	Reference
Protein	CA125	116 OC, 304 BeC	ATR-FTIR s	NR	No chemo: 29 Chemo: 57	No chemo: 87 Chemo: 92	[219]
Proteins	HE4, CA125, OPN	51 OC, 34 other cancers, 14 BeC, 33 H	pGOLD chips	HE4: 0.85 (0.78–0.93); CA125: 0.75 (0.67–0.84); OPN: 0.77 (0.67–0.86)	90.2 (HE4, 72.55; CA125, 58.82; OPN, 68.63)	80.25 (HE4, 95.06; CA125, 87.65; OPN, 90.12)	[220]
Metabolites	L-pyroglutamic acid, turanose, glycine, L-threonine, L-tyrosine (OC vs. BeC)	29 OC, 24 BeC, 22 H	GC-MS	OC v BeC: 0.88 (0.74–0.96); OC v H: 0.98 (0.96–1); BeC v H: 0.98 (0.96–1)	OC v BeC 90.72; OC v H 93.81; BeC v H 97.01	OC v BeC 82.09; OC v H 98.59; BeC v H 97.18	[222]
Protein	MCM5	26 OC, 41 EC, 58 BeC	ELISA	OC vs. BeC: 0.68	OC vs. BeC: 61.5; Low grade: 71.4 (29.0–96.3); low stage: 75 (34.9–96.8)	OC vs. BeC: 75.9 (62.8–86.1)	[224]
Proteins	WFDC2, PTMA, PVRL4, FIBA, PVRL2	73 OC, 59 BeC	DIA-MS	0.96	0.87	0.91	[225]
Metabolites	metabolic profile	150 OC, 81 BeC, 20 borderline, 80 H	UHPLC-QTOF-MS	Training: H v OC, 0.98; BeC v OC, 0.87; borderline v OC, 0.94. Validation: 0.98, 0.89, 0.83, respectively	Training: H v OC, 94.2; BeC v OC, 87.36; borderline v OC, 98.65. Validation: 97.66, 86.36, 80.26, respectively	Training: H v OC, 95; BeC v OC, 62.86; borderline v OC, 84.62. Validation: 97.59, 78.57, 71.43, respectively	[223]
Proteins	CA125, HE4	17 OC, 51 BeC, 46 H	CLEIA	CA125: 0.69 (0.55–0.82); HE4: 0.71 (0.60–0.82); Combined: 0.71 (0.60–0.82). Early Stage- Ca125: 0.67 (0.51–0.83), HE4: 0.68 (0.56–0.81)	CA125: 64.7 (38.2–85.8); HE4: 70.6 (44.0–89.7); Combined: 82.4 (56.6–96.2). Early Stage: 76.9 (46.2–95.0)	CA125: 74.2 (64.3–82.6); HE4: 58.8 (48.3–68.7); Combined: 40.2 (30.4–50.7)	[221]
VOCs	VOC panel	50 OC; 43 BeC; subanalysis 31 OC, 29 BeC	electronic nose and tongue (liquid sensor array)	NR	86; e tongue: 78	88; e tongue: 53	[226]
Proteins	23 biomarkers	158 OC, 125 BeC	xMAP bead-based multiplexed immunoassay	HE4 + Cr + CEA + TTR: 0.938	HE4 + Cr + CEA + TTR: 93.7	HE4 + Cr + CEA + TTR: 70.6	[227]
Protein	P-LAP	18 OC, BeC 22	LAP assay kit	NR	77.8	95.5	[228]

BeC, benign control; EC, endometrial cancer; H, healthy; NR, not recorded.

### 3.11. Existing Technologies (Bladder and Prostate Cancers)

Bladder (BCa) and prostate (PCa) cancers were excluded from the detailed literature search because several urine-based assays for their detection and clinical management have already achieved clinical translation and are commercially available and/or routinely used in clinical practice. Therefore, only a brief overview of these malignancies is provided, together with a summary of currently available urine-based tests in Table 11.

Bladder cancer is one of the most common urological malignancies, ranking among the ten most frequently diagnosed cancers worldwide, with over 613,000 new cases reported in 2022 [1]. In the UK, around 10,900 new cases are diagnosed annually [229]. Owing to its high recurrence rate and the requirement for lifelong surveillance, considerable effort has been devoted to the development of non-invasive urinary biomarkers to complement cystoscopy. Several urine-based assays have been approved by the U.S. Food and Drug Administration (FDA), including UroVysion FISH, which detects chromosomal abnormalities involving chromosomes 3, 7, and 17, as well as deletion of the 9p21 locus; NMP22 BladderChek and BTA stat/TRAK, which measure tumour-associated proteins; and uCyt+, which detects tumour-associated cell surface antigens. More recently, molecular assays have expanded the diagnostic landscape, including Cxbladder, a urinary mRNA expression panel; AssureMDx, which combines FGFR3, TERT and HRAS mutation analysis with DNA methylation markers; Xpert Bladder Cancer Monitor, a five-gene mRNA assay; UroMuTERT, which detects TERT promoter mutations; and Bladder EpiCheck, a 15-marker DNA methylation panel. These tests have demonstrated utility in the non-invasive detection and surveillance of bladder cancer and support a more personalised approach to patient management [230,231].

Prostate cancer is the second most commonly diagnosed cancer in men worldwide, with approximately 1.47 million new cases and 397,000 deaths reported in 2022, making it the fifth leading cause of cancer-related mortality among men globally [232]. In the UK, it is the most frequently diagnosed male malignancy, accounting for approximately 55,000 new cases and more than 12,000 deaths annually [233]. Despite its high incidence, over half of screen-detected, early-stage tumours are considered indolent and unlikely to cause clinical harm during a patient’s lifetime [234,235], presenting a major challenge for population-based screening programmes. The widespread adoption of prostate-specific antigen (PSA) testing in the early 1990s substantially increased the detection of asymptomatic disease but also led to considerable overdiagnosis and overtreatment, with estimates suggesting that 20–50% of screen-detected cancers may represent overdiagnosis [234,236]. These limitations have highlighted the need for biomarkers that can more accurately distinguish clinically insignificant disease from aggressive, clinically significant prostate cancer (csPCa).

Several urine-based assays have been developed to address this unmet need. SelectMDx combines the urinary expression of HOXC6 and DLX1 mRNA with clinical risk factors to predict the presence of csPCa, achieving reported negative predictive values of 82–95% for high-grade disease [237,238]. ExoDx Prostate IntelliScore (EPI) quantifies exosomal RNA biomarkers (PCA3, ERG, and SPDEF) without requiring a digital rectal examination and has demonstrated an AUC of approximately 0.77 for the detection of Grade Group ≥ 2 cancer at initial biopsy [239]. The FDA-approved PCA3 (PROGENSA) assay is used to support repeat-biopsy decision-making in men with elevated PSA levels and a previous negative biopsy, with reported AUC values ranging from 0.68 to 0.87 [240,241]. More recently, MyProstateScore 2.0 (MPS2), which incorporates TMPRSS2:ERG fusion gene, PCA3 and PSA, demonstrated approximately 97% SN for ruling out Grade Group ≥ 2 prostate cancer in validation cohorts [242].

Collectively, these assays exemplify the transition towards more precise, biology-driven risk stratification in prostate cancer. By improving the identification of clinically significant disease while reducing unnecessary biopsies and the detection of indolent tumours, urinary biomarkers have the potential to support more personalised and effective prostate cancer management.

## 4. Discussion

Here, we reviewed studies reporting the discovery and validation of urinary biomarkers for the early detection of multiple cancer types, with the aim of assessing both the progress made and the challenges that remain in this field.

One such challenge became evident during the literature search. Although our search strategy retrieved a large number of publications, only a small proportion met the predefined inclusion criteria. This highlights the issues associated with literature retrieval in rapidly expanding research fields, even when employing comprehensive search strategies that combine MeSH terms with free-text keywords. Despite filtering by publication date, language, study type, and topic, many retrieved articles remained irrelevant to the objectives of this review. Of the 10,512 records identified across the three databases, only approximately 1.5% were ultimately eligible for inclusion. This finding is consistent with the observations of Leblanc et al., who reported that combining MeSH terms with free-text keywords increases the number of studies requiring screening but may improve the identification of relevant publications compared with the use of MeSH terms alone [243]. While this approach inevitably increases the literature search burden, it is reassuring that it enhances the sensitivity of the search and thus reduces the likelihood of missing eligible studies.

Notably, clear differences were observed among the databases searched. Embase yielded substantially more records than either PubMed or Web of Science, reflecting differences in indexing practices, database coverage, and search algorithms. However, the higher volume of retrieved records did not necessarily translate into a proportionally greater number of eligible studies. These findings emphasise the difficulties of developing search strategies that are both sensitive and specific, as well as the importance of searching multiple databases when conducting comprehensive literature reviews.

The recent advances in artificial intelligence (AI) and machine learning have the potential to improve the prioritisation and screening of publications from increasingly large literature datasets. Their impact on accurate selection and potential introduction of selection bias, however, remains an area of ongoing evaluation. Consequently, this scoping review employed conventional manual screening to ensure transparent and consistent application of the predefined inclusion and exclusion criteria across different cancer and analyte types. Importantly, although the majority of the retrieved records were excluded, the overall volume of publications identified demonstrates the growing research interest in urinary biomarkers and highlights the rapid development of this field.

This increase in research output has unfortunately not been matched by equivalent progress towards clinical implementation. While a substantial number of publications were reviewed, clinical readiness of most urinary biomarkers remains limited. Key diagnostic performance measures, including AUC, SN and SP were inconsistently reported, while PPV and NPV were available in only a minority of studies. Another common limitation was the use of relatively small patient cohorts, precluding the incorporation of separate training and validation sets that are needed to minimise overfitting and provide reliable estimates of diagnostic performance. A further challenge observed across several studies was the frequent use of healthy controls rather than clinically relevant benign disease controls, which may overestimate the reported SNs and SPs, and does not reflect the real-world diagnostic setting, where the key challenge is distinguishing cancer from benign conditions with similar clinical presentations. This limitation may also contribute, at least in part, to some of the exceptionally high AUC values reported in the reviewed literature.

Therefore, at present, much of the published literature remains exploratory with reported biomarkers being at the discovery stage and insufficiently validated for clinical application. Overall, these findings highlight the need for larger, well-designed validation studies that use both clinically relevant controls and comprehensive reporting of diagnostic performance metrics.

Translating promising urinary biomarkers into clinical practice remains challenging because, despite its many advantages, urine is subject to several biological and technical limitations. Standardisation of pre-analytical variables, including collection methods (e.g., first-void versus mid-stream urine) and timing of collection, sample processing, and storage conditions, remains a major unmet need and continues to contribute to variability between studies [244,245]. Notably, since the comprehensive systematic review of preanalytical parameters and applied methodologies in urine biomarker research by Jordaens et al. [13], there has been limited improvement in the reporting of these parameters.

Additional challenge, particularly evident in metabolomics studies, the most frequently represented biomarker class in this review, was analytical variability, which can substantially influence biomarker discovery and performance estimates. Although several studies reported the use of internal standards, quality-control samples and quality-assurance procedures, their implementation and reporting were inconsistent. Information regarding analytical drift monitoring, batch-effect correction, and metabolite identification criteria was often limited or absent, making it difficult to assess reproducibility across studies. Inadequate reporting and a lack of standardisation of data normalisation methods thus remain prevalent, despite being previously highlighted as a key limitation by Nam et al. [246].

Urinary biomarker measurements may also be influenced by physiological and environmental factors such as age, sex, diet, hydration status, medication use, and comorbidities including diabetes, hypertension, and urinary tract infections, highlighting the need for future studies to incorporate comprehensive clinical and demographic data, together with appropriate statistical adjustment for potential confounders. In addition, changes in renal function can affect biomarker concentration, excretion, and stability, necessitating careful consideration of kidney disease during biomarker development and validation. Certain analytes, particularly circulating tumour DNA (ctDNA) and microRNAs (miRNAs), may be present at lower concentrations in urine than in blood or organ-proximal fluids, posing additional challenges for early cancer detection where tumour burden is low. To address these issues and improve reproducibility across studies, a welcome recent development is the proposed MUMIE (Minimal Urine Methods in Experiments) framework, which aims to standardise urine collection, processing, and reporting practices [247].

Nevertheless, accumulating evidence supports the biological relevance of urinary biomarkers. Zhang et al. demonstrated that urinary DNA profiles could be matched to corresponding tumour tissue, confirming that urine contains tumour-derived molecular information [248]. Together with ongoing advances in assay sensitivity, sample processing, and biomarker validation, these findings reinforce the considerable potential of urine as a biofluid for future cancer early detection strategies.

### The Future

As demonstrated in this review, despite the aforementioned challenges, urine-based liquid biopsy has considerable potential to transform cancer detection and diagnosis. Given the biological complexity and heterogeneity of cancer, it is unlikely that a single biomarker or sample type will provide sufficient diagnostic accuracy across all settings. Instead, future diagnostic strategies are likely to rely on multi-analyte and multimodal approaches [249,250]. As these approaches are likely to increase testing costs, health economic analyses will be required to determine whether improved diagnostic performance offsets the additional expenditure. Being non-invasive, inexpensive, and easily repeatable, the incorporation of urine testing into existing diagnostic pathways may be particularly advantageous. Urinary biomarkers may be especially valuable as a first-line triage tool, identifying individuals who would benefit from further investigations while reducing unnecessary testing in low-risk populations, and could help reduce the need for more invasive procedures or costly radiological examinations in selected patient groups.

Of note, many of the studies excluded from this review, due to the focus on diagnosis, also highlighted the broader utility of urinary biomarkers—beyond early detection, urine-based assays show promise for disease monitoring, prediction of recurrence, assessment of prognosis, and evaluation of treatment response.

Perhaps one of the most exciting future applications of urinary biomarkers is their potential to enable home-based and community-based cancer testing. Because urine can be collected non-invasively without the need for a clinical visit, it lends itself to remote screening and longitudinal monitoring. Advances in biosensors, point-of-care diagnostics, and lateral flow technologies are increasingly supporting rapid, user-friendly testing outside traditional healthcare settings [251].

Such approaches could improve participation in cancer screening programmes by reducing the inconvenience and invasiveness associated with current screening methods, while expanding access to early detection and personalised disease monitoring. The growing emphasis on accessible, patient-centred diagnostics and the integration of liquid biopsy technologies into screening pathways further support this vision for the future.

Recent technological developments further support this vision. Smart toilet systems capable of automatically collecting and analysing urine are now being actively developed, creating opportunities for continuous, passive health monitoring without requiring any change in user behaviour. These platforms integrate urine sampling with sensors, imaging, and digital health technologies, potentially enabling early disease detection, longitudinal monitoring, and seamless integration into future precision medicine pathways [252]. AI is likely to play an increasingly important role in this evolving landscape. AI-driven algorithms could assist with the interpretation of complex multi-analyte data, integration of results from multiple biofluids, and generation of personalised risk assessments. When combined with home-based testing platforms, AI-enabled systems may allow real-time result interpretation, monitoring of longitudinal trends, and rapid communication with healthcare providers. However, successful implementation will require careful attention to regulatory oversight, data security, patient privacy, and management of false-positive results.

Finally, large-scale initiatives such as the World Urine Project aim to systematically characterise the urinary biomarker landscape across health and disease through international collaboration and the development of shared data resources [253]. By generating comprehensive reference datasets and promoting standardised research approaches, such initiatives will provide a critical foundation for biomarker discovery, validation, and clinical translation, thereby accelerating the future development of urine-based diagnostics [253].

## 5. Conclusions

In summary, the evidence reviewed here highlights the considerable potential of urinary biomarkers to transform cancer detection and management. Although important challenges, including the need for greater standardisation of pre-analytical and analytical procedures, robust validation in large prospective cohorts, and demonstration of clinical utility across diverse populations remain, advances in biomarker discovery, multi-omics technologies, AI and home-based testing are rapidly accelerating progress towards future clinical translation. With further validation and standardisation, urine-based diagnostics may become an important component of precision oncology, enabling earlier detection, and ultimately better patient outcomes.

## Figures and Tables

**Table 1:** Summary of database output and relevant papers by database.

Cancer	Embase		PubMed		Web of Science		Final Number *
	Output	Relevant	Output	Relevant	Output	Relevant	
Breast Cancer	444	41	258	52	322	40	25
Lung Cancer	2518	10	248	14	342	14	16
Gastric Cancer	162	32	237	55	51	25	10
Hepatocellular Carcinoma	313	14	524	11	180	8	13
Cholangiocarcinoma	192	2	18	2	22	1	5
Pancreatic Cancer	266	22	148	12	148	12	20
Colorectal Cancer	217	10	258	11	409	15	16
Renal Cell Carcinoma	629	33	95	1	501	31	41
Ovarian Cancer	221	20	218	18	126	20	10
Ampullary Cancer	18	0	1	0	1	0	0
Duodenal Cancer	1265	0	148	0	12	0	0

* Excluding duplicate papers.

**Table 4:** Urinary biomarkers in gastric cancer (GC).

Biomarker Type	Biomarker(s)	Study Population	Method	AUC (95% CI)	SN (95% CI)	SP (95% CI)	Reference
miRNAs	miR-6807-5p/miR-6856-5p/*H. pylori* status	Discovery: 4 GC, 4 H Training: 95 CG, 95 H Validation: 54 GC, 54 H	Agilent Human miRNA Microarrays and qPCR	Validation: 0.88 (0.82–0.95); Stage I: 0.75 (0.68–0.81)	Validation, GC: 76.9; Stage I GC: NR	Validation, GC: 88.90; Stage I GC: NR	[70]
DNA	8-OHdG and 8-OHG (oxidative modification/damage by ROS)	60 GC, 70 H	UPLC-MS/MS	0.77 (0.69–0.85; 0.84 (0.77–0.91)	NR	NR	[75]
Metabolites	Nine metabolites (alanine, citrate, creatine, creatinine, glycerol, hippurate, phenylalanine, taurine, 3-hydroxybutyrate)	Training: 69 GC, 67 H Validation: 34 GC, 33 H	NMR	NR	All Stages: 93.9; Stage IA: 94.7	All Stages: 94.1; Stage IA: 97	[74]
*C. elegans* VOCs	VOCs	180 GC, 76 H	N-NOSE	0.86 (0.81–0.91)	NR	NR	[76]
Bile acids	Hyodeoxycholic acid, cholic acid, glycochenodeoxycholic acid, chenodeoxycholic acid	76 GC, 32 H	LC-MS/MS	0.86, 0.85, 0.75, 0.77, respectively	NR	NR	[77]
Microbiome	EV microbiome panel	181 GC, 272 H	NGS (16s rRNA MiSeq)	0.82	67	85	[78]
Amino-containing metabolites	β-(Pyrazol-1-yl)-L-alanine (L-PA), D-Ser and D-Ile	Training: 43 GC, 40 H Validation: 41 GC, 40 H	HPLC-MS/MS	0.88 (0.82–0.95), 0.78 (0.67–0.87), 0.76 (0.65–0.86), respectively. Combined with age: 0.97 (0.95–1.00)	83, 58, 58, respectively. Combined: 90	77, 87, 95, respectively. Combined: 95	[72]
Proteins	uTFF1/uADAM12/*H. pylori* status (males); uTFF1/uBARD1/*H. pylori* status (females)	Discovery: 12 GC, 6 H Training: 88 GC, 86 H Validation: 44 GC, 44 H	LC-MS/MS ELISA	Stage I males: 0.85 (0.78–0.91); Stage I females: 0.84 (0.74–0.95)	Stage I males: 91.5; Stage I females: 100	Stage I males: 52.5; Stage I females: 51.6	[71]
Proteins	ANXA11, CDC42, NAPA, SLC25A4	Training: 14 GC, 109 BeC; Validation: 18 GC, 114 BeC	LC-MS/MS	For predicting progression: 0.92 (0.83–1.00); risk: 0.81 (0.73–0.89)	NR	NR	[79]
Proteins	SORT1, VTN	Training: 5 GC, 5 H Validation: 19 GC, 12 H	LC-MS/MS	SORT1: 0.82; VTN: 0.78. Combined: 0.83	NR	NR	[80]

H, healthy; NR, not recorded.

**Table 5:** Urinary biomarkers in hepatocellular carcinoma (HCC).

Biomarker Type	Biomarker(s)	Study Population	Method	AUC (95% CI)	SN% (95% CI)	SP% (95% CI)	Reference
Protein	NGAL	40 HCC, 40 LC, 40 chronic HBV or HCV, 40 H	ELISA	HCC v LC: NGAL 0.95, NGAL + serum AFP 0.99	HCC v LC: NGAL 90, NGAL + serum AFP 95	HCC v LC: NGAL 87.5, NGAL + serum AFP 100	[91]
Proteins	u-AFP, u-ORM1	Training: 10 HCC, 10 CHB, 10 LC, 10 H Validation: 75 HCC, 51 LC 14 CHB	LC-MS/MS; ELISA	HCC v non-HCC: u-AFP 0.79 (0.70–0.88); u-ORM1 0.70 (0.60–0.80); u-AFP + u-ORM1 0.86 (0.79–0.93)	HCC v non-HCC: u-AFP 63.5; u-ORM1 59.6; u-AFP + u-ORM1 80.9	HCC v non-HCC: u-AFP 95.4; u-ORM1 77.4; u-AFP + u-ORM1 85.5	[94]
Proteins	7 peptides: HPX, APOH, APCS, PLG, GOT1, GLRX, NCR3LG1	Training: 36 HCC, 29 LC, 14 CHB Cross-validation: 18 HCC, 10 LC, 9 CHB External validation: 20 HCC, 15 LC, 5 CHB	LC-MS/MS; RF	HCC v non-HCC: RF cross-validation 0.92, test set 0.87; voting algorithm cross-validation 0.94, test set 0.90; MRM 0.95	HCC v non-HCC: RF cross-validation 86.1; voting algorithm 100; MRM 90	HCC v non-HCC: RF cross-validation 86; voting algorithm 78.9; MRM 85	[95]
Peptides	31 peptides mapped on 7 proteases: KLK6, MMP-3, MMP-13, CTSD, CTSE, MEP1A, CTSB	Training: 18 HCC, 51 BeC Validation: 39 HCC, 87 BeC	CE-MS/MS; IHC; (SVM model)	Validation: 0.88 (0.81–0.93)	Validation: 79.5	Validation: 85.1 (0.81–0.93)	[96]
VOCs	VOCs (not specified)	31 HCC, 62 PCa, 29 BCa, 18 BeC	SPME; PCA, RBFN	AFP ≥ 10 kU/L: 0.83 (0.73–0.93); in AFP ≤ 10 kU/L: 0.68 (0.54–0.81)	AFP ≥ 10 kU/L: 83 (0.73–0.93); in AFP ≤ 10 kU/L: 68 (0.54–0.81)	NR	[97]
VOCs	VOC profile; 7 VOCs	20 HCC, 38 NAFLD with and without fibrosis	GC-IMS, GC-TOF-MS/MS; RF, LR	GC-IMS + RF: HCC v fibrosis 0.97 (0.91–1.00); HCC v non-fibrosis 0.62 (0.48–0.76) GC-TOF-MS + LR: HCC v fibrosis 0.79 (0.50–1.00); HCC v non-fibrosis 0.83 (0.62–1.00)	GC-IMS + RF: HCC v fibrosis 43 (0.13–0.75); HCC v non-fibrosis 60 (0.41–0.78) GC-TOF-MS + LR: HCC v fibrosis 71 (0.40–1.00); HCC v non-fibrosis 66 (0.33–1.00)	GC-IMS + RF: HCC v fibrosis 95 (0.86–1.00); HCC v non-fibrosis 74 (0.61–0.87) GC-TOF-MS + LR: HCC v fibrosis 66 (0.33–1.00); HCC v non-fibrosis 71 (0.43–1.00)	[98]
Microbiome	16S rRNA	Training: 471 HCC, 397 H Validation: 164 HCC, 164 H	Metagenomic sequencing	Training: 0.89; Validation: 0.94	Validation: 0.89	Validation: 0.88	[99]
Metabolites	adenine, alanine, creatinine, deoxyribose, guanine, histidine, proline, serine, tryptophan, uracil, urea, uric acid, others	55 HCC, 49 LC, 50 H	SERS	Accuracy: HCC v LC 0.78; HCC + LC v H 0.87; HCC v H 0.85	HCC v LC 79.6; HCC + LC v H 92; HCC v H 85.5	HCC v LC 76; HCC + LC v H 77.8; HCC v H 84	[100]
Metabolites	porphyrin derivatives and bilirubin	62 HCC, 65 LC, 60 H	Urine fluorescence spectroscopy at 405nm	Accuracy: HCC v H 0.96; LC v H 0.91; HCC v LC 0.86; HCC + LC v H 0.93	HCC v H 100; LC v H 95.4; HCC v LC 93.55; HCC + LC v H 93.2	HCC v H 91.7; LC v H 86.7; HCC v LC 80; HCC + LC v H 94.4	[101]
Metabolites	Six metabolites	Training: 104 HR, 147 HCC, 155 H Validation: 44 BeC, 70 HCC, 61 H	MS	Validation: HCC v HR 0.84 (0.64–0.86); HCC v H 0.96 (0.91–0.96)	NR	NR	[90]
cfDNA	TP53 249T, mRASSF1a, mGSTP1 ± serum AFP	186 HCC, 279 HBV, 144 LC	qMSP assay	HCC v non-HCC ctDNA 0.74 (0.70–0.78); ctDNA + serum AFP 0.91	HCC v non-HCC ctDNA + serum AFP 79.6, Stage 0: 92, Stage A: 77	HCC v non-HCC ctDNA + serum AFP 90	[93]
circular RNA (circRNA)	Panel of three: circ_0075792, circ_0005397, circ_0000976 (selected from literature)	Discovery: 50 CHB, 50 LC, 22 HCC Stage I/II, 51 HCC, 50 H Training: 73 CHB, 69 LC, 42 HCC Stage I/II, 85 HCC, 85 H Validation: 174 CHB, 178 LC, 93 HCC Stage I/II, 196 HCC, 193 H	RT-qPCR + LR	Training: HCC Stage I/II v H 0.92; HCC Stage I/II v CHB 0.92; HCC Stage I/II v LC 0.92; HCC v H 0.94; HCC v CHB 0.92; HCC v LC 0.92. Validation: HCC Stage I/II v H 0.93; HCC Stage I/II v CHB 0.91; HCC Stage I/II v LC 0.93; HCC v H 0.96; HCC v CHB 0.95; HCC v LC 0.96	Training: HCC Stage I/II v H 82.5; HCC Stage I/II v CHB 90.1; HCC Stage I/II v LC 90.8; HCC v H 90.9; HCC v CHB 87.1; HCC v LC 90.6. Validation: HCC Stage I/II v H 90; HCC St I/II v CHB 90.5; HCC St I/II v LC 80.5; HCC v H 90.8; HCC v CHB 87.2; HCC v LC 91.8	Training: HCC Stage I/II v H 95.2; HCC Stage I/II v CHB 85.5; HCC Stage I/II v LC 85.1; HCC v H 99.1; HCC v CHB 97.3; HCC v LC 98.6. Validation: HCC Stage I/II v H 90; HCC Stage I/II v CHB 90.8; HCC Stage I/II v LC 100; HCC v H 99; HCC v CHB 99.4; HCC v LC 99.4	[102]
miRNAs	miR-93–5p	64 early HBV-related HCC (Stages IA, IB, IIA); 66 advanced HBV-related HCC; 65 H	RT-qPCR, expression analysis (GEO2R)	Early HBV-related HCC v H: 0.90; advanced HCC v H: 0.92; HCC v H: 0.91	Early HBV-related HCC v H: 87.5; advanced HCC v H: 87.9; HCC v H: 87.7	Early HBV-related HCC v H: 95.4; advanced HCC v H: 95.4; HCC v H: 95.4	[92]

BCa, bladder cancer; BeC, benign controls; CHB, chronic hepatitis B; cfDNA, cell free DNA; HBV, hepatitis B virus; HCV, hepatitis C virus; H, healthy; LC, liver cirrhosis; LR, logistic regression; H, healthy; HR, high-risk; IHC, immunohistochemistry, m, methylated; MRM, multiple reaction monitoring; NAFLD, non-alcoholic fatty liver disease; NR, not recorded; PCa, prostate cancer; PCA, principal component analysis; RBFN, radial basis function networks; RF, random forest; St, stage; SVM, support vector machine; u-AFP, urinary α-fetoprotein; u-ORM1, urinary orosomucoid 1.

**Table 6:** Urinary biomarkers in cholangiocarcinoma (CCA).

Biomarker Type	Biomarker(s)	Study Population	Method	AUC (95% CI)	SN (95% CI)	SP (95% CI)	Reference
Metabolites	CR, NANA, CS, Met 561+ and CA19.9	117 HCC, 228 BeC, 253 ICC, 243 ICC BeC, 251 H	UPLC/MS-MS	ICC v ICC BeC: 0.88 (0.85–0.91)	ICC v ICC BeC: 71	ICC v ICC BeC: 92	[113]
EVs	INO80D, MAP6D1, RRAGD, HCG4, miR200C, LOC100134868; CLIP3, VCAM1, TRIM33, ATP5EP2, LOC100134713, SNORA8	23 CCA, 5 H (CCA vs. H); 23 CCA, 5 PSC (CCA vs. PSC)	RNA microarray	1.00, 1.00, 1.00, 0.93 (0.83–1.00), 0.90 (0.74–1.00), 0.90 (0.77–1.00); 0.97 (0.90–1.00), 0.97 (0.90–1.00), 0.97 (0.90–1.00), 0.94 (0.85–1.00), 0.93 (0.83–1.00), 0.92 (0.82–1.00)	100, 100, 100, 87, 95.70, 87; 87, 87, 87, 87, 82.60, 82.60	100, 100, 100, 100, 80, 100; 100, 100, 100, 100, 100, 100	[105]
Metabolite	8-oxodG	20 H, 24 *O. viverrini* infected (4 PDF +ve, 20 PDF -ve)	ELISA	0.90	75	97.50	[115]
Protein	*O. viverrini* antigen	243 *O. viverrini +ve* and 250 *O. viverrini −ve*	RDT and ELISA	NR	OV-RDT: 94.20; ELISA: 100	OV-RDT: 93.20; ELISA: 93.60	[114]
EVs	PLA2G2A and PLA2G12B	CCA, non-tumour	Bioinformatic analysis	0.88; 0.47	NR	NR	[104]

CR, creatine riboside; CS, cortisol sulfate; CCA, cholangiocarcinoma; H, healthy; Met 561+, lipid metabolite 561+; NANA, N-acetylneuraminic acid; NR, not recorded; OV, *Opisthorchis viverrini*; OV-RDT, Opisthorchis viverrine-rapid diagnosis test; PDF, periductal fibrosis; PSC, primary sclerosing cholangitis; RDT, rapid diagnosis test.

**Table 8:** Urinary biomarkers in colorectal cancer (CRC).

Biomarker Type	Biomarker(s)	Study Population	Method	AUC (95% CI)	SN (95% CI)	SP (95% CI)	Reference
Metabolites	17 metabolites	171 CRC, 171 H	LC-MS	0.86	80.00	80.00	[149]
Aminoacids	D-AABA, D-Ala, D-Gln, and D-Ser	115 CRC, 155 H	LC-MS	0.99	98.30	96.80	[150]
VOCs	VOC profiles	12 CRC, 80 adenoma, 33 BeC, 37 H	FAIMS, GC–IMS	CRC v H (FAIMS): 0.98 (0.93–1.0); CRC vs. adenoma (GC-IMS): 0.92 (0.77–1.0)	0.83 (0.52–0.98)	1 (0.59–1)	[158]
Proteins	CORO1C, RAD23B, ARPC5	263 CRC, 289 H	TMT labeling-2D-LC-MS/MS	0.80	75.80	88.00	[161]
Metabolites	N1, N12-diacetylspermine, hippurate, -hydroxyhippurate and glutamate	212 CRC, 77 BeC, 225 H	LC-MS/MS	Training: 89.2 Validation: 89.7	83.43	79.38	[151]
Proteins	DPEP1 and TFF1	Discovery: 16 CRC, 16 H Training: 110 CRC, 110 H Validation: 49 CRC, 62 H	LC–MS/MS, ELISA	Training: 0.82; Validation: 0.96	Stage 0/I CRC: 93.8	Stage 0/I CRC: 63.4	[153]
Metabolites	TAU, AL and BAIB	92 CRC (36 advanced), 156 H	NMR	TAU: 0.82; ALA: 0.78; BAIB: 0.84	96	95	[152]
DNA methylation	SEPT9, TMEFF2, SDC2, NDRG4, VIM, ALX4	92 CRC, 63 H	qMSP	0.78	70	86	[154]
Proteins	uCyr61, uTFF3	Training: 53 CRC, 53 H Validation: 35 CRC, 35 H	ELISA	Training: uCyr61, 0.74 (0.65–0.84); uTFF3, 0.75 (0.46–0.68); uCyr61 + uTFF3, 0.75 (0.66–0.85) Validation: uCyr61, 0.69 (0.57–0.82); uTFF3: 0.64 (0.51–0.77); uCyr61 + uTFF3, 0.72 (0.56–0.84)	Training: uCyr61 or uTFF3, 75.5 Validation: uCyr61 or uTFF3, 71.4	Training: uCyr61 or uTFF3, 69.8 Validation: uCyr61 or uTFF3, 74.3	[162]
miRNA	miR-129-1- 3p and miR-566	Discovery: 3 CRC, 6 H Training: 140 CRC, 140 H Validation: 63 CRC, 63 H	qRT-PCR	Training: miR-129-1- 3p, 0.79 (0.74–0.84); miR-566, 0.72 (0.66–0.78); Combined: 0.811 (0.76–0.86); Validation: miR-129-1- 3p, 0.85 (0.79–0.92); miR-566, 0.80 (0.73–0.88); Combined: 0.87 (0.80–0.93); Stage 0/I, 0.84 (0.79–0.89)	Combined, Training: 80.7; Validation: 88.9; Stage 0/I, 82.8	Combined, Training: 70.7; Validation: 76.2	[155]
Metabolites	Upregulation of glycolysis and amino acid metabolism	163 CRC, 111 H	GC-TOF/MS	NR	98.70	83.30	[163]
DNA/RNA/Protein Lipid	NR	63 CRC, 53 H	SERS	Stages I/II CRC: 0.95; Stages III/IV CRC: 0.96	80.2 84.3	95 94.2	[159]
VOCs	VOCs profiles	18 CRC, 31 CRC polyps, 34 H	GC-MS	CRC and polyps v H: 0.89 (0.80, 0.96)	CRC and polyps v H: 0.88 (0.75, 0.95)	CRC and polyps v H: 0.88 (0.72, 0.96)	[156]
Proteins	MMPs 2, 7 and 9	82 CRC, 47 CRC polyps, 59 H	Luminescence	CRC v H: 0.97 (0.96–0.99); early CRC v H, 0.97 (0.94–1.00)	CRC v H: 95.10; early CRC v H, 94.30	CRC v H: 91.50; early CRC v H, 98.30	[160]
RNA	8-OHG	65 CRC, 76 H	UPLC-MS/MS	0.75 (0.67–0.83)	NR	NR	[164]
VOCs	VOCs profiles	56 CRC, 82 H	FAIMS	0.71 (0.62–0.8)	69 (54–81)	69 (57–79)	[157]

H, healthy; NR, not recorded.

**Table 11:** Approved Urine-Based Tests for Bladder and Prostate Cancers.

Test Name	Cancer	Biomarker Type	Regulatory Status	Notes
UroVysion FISH	Bladder	Chromosomal abnormalities	FDA approved	Detects aneuploidy of chromosomes 3, 7, 17 and deletion of 9p21
NMP22 BladderChek	Bladder	Nuclear matrix protein	FDA approved	Point-of-care test; detects NMP22 protein elevated in bladder cancer
BTA stat/BTA TRAK	Bladder	Human complement factor H-related protein	FDA approved	BTA stat: point-of-care; BTA TRAK: quantitative ELISA
Cxbladder	Bladder	mRNA expression (gene panel)	FDA approved/CE-marked Available in several countries	High sensitivity; used for diagnosis and surveillance
AssureMDx	Bladder	DNA mutations and methylation	CLIA-lab validated (Irvine, CA, USA)	Detects FGFR3, TERT, and HRAS mutations
Xpert Bladder Cancer Monitor	Bladder	mRNA expression (5-gene panel)	CE-marked	Used for recurrence monitoring
uCyt+	Bladder	Cell surface antigens (immunofluorescence)	FDA approved	Detects mucin and CEA-related antigens
UroMuTERT	Bladder	TERT promoter mutations in uDNA	NR	Sensitive for early-stage disease
EpiCheck	Bladder	DNA methylation markers (15-marker panel)	CE-marked, CLIA-lab validated (USA)	Used for recurrence monitoring
SelectMDx, Confirm MDx	Prostate	mRNA biomarkers (DLX1, HOXC6)	CE-marked	Aids biopsy decision-making; risk stratification
ExoDx Prostate IntelliScore (EPI)	Prostate	Exosomal RNA (ERG, PCA3, SPDEF)	CLIA-lab validated (USA)	Predicts high-grade prostate cancer
PCA3 (Progensa)	Prostate	Non-coding mRNA (PCA3)	FDA approved	Aids biopsy decisions
MyProstate Score (MPS)	Prostate	TMPRSS2:ERG fusion gene + PCA3 + PSA	CLIA-lab validated (USA)	Combines multiple biomarkers to improve risk assessment for aggressive prostate cancer
Proclarix	Prostate	Thrombospondin-1 (THBS1), cathepsin D (CTSD), total PSA (tPSA), and free-PSA (%fPSA)	CE-marked	Assesses the risk of aggressive prostate cancer

NR, not recorded.

## Data Availability

The original data presented in the study are openly available in the manuscript. These data were derived from the following resources available in the public domain: Embase https://www.embase.com/search/quick (accessed on 9 October 2026), PubMed https://pubmed.ncbi.nlm.nih.gov/ (accessed on 9 October 2026) and Web of Science https://www.webofscience.com/wos/woscc/smart-search (accessed on 9 October 2026).

## Supplementary Materials

The following supporting information can be downloaded at: https://www.mdpi.com/article/10.3390/cancers18203328/s1, Table S1: Preferred Reporting Items for Systematic reviews and Meta-Analyses extension for Scoping Reviews (PRISMA-ScR) Checklist.

## Abbreviations

The following abbreviations are used in this manuscript: Platforms and methods

AuNPET	gold nanoparticle enhanced target
AC	Affinity chromatography
ATR-FTIR	Attenuated Total Reflectance Fourier Transform Infrared Spectroscopy
BS-qPCR	bisulfite quantitative PCR
CE-MS/MS	capillary electrophoresis mass spectrometry
cfMeDIP-seq	cell-free methylated DNA immunoprecipitation and high-throughput sequencing
CMIA	chemiluminescent micro particle immunoassay
DIA-MS	Data-independent acquisition-mass spectrometry
1D SDS-PAGE	1-dimensional Sodium dodecyl-sulfate polyacrylamide gel electrophoresis
ECLIA	electro chemiluminescent immunoassay
ELISA	enzyme-linked immunosorbent assay
FAIMS	field asymmetric waveform ion mobility spectrometry
GC	gas chromatography
GC-HRMS	gas chromatography-high resolution mass spectrometry
GC-IMS	gas chromatography–ion mobility spectrometry
GC-TOF-MS/MS	gas chromatography–time-of-flight mass spectrometry
HILIC-MS/MS	hydrophilic interaction liquid chromatography-tandem mass spectrometry
^1^H-NMR	proton nuclear magnetic resonance
HPPI-TOFMS	high-pressure photon ionization time-of-flight mass spectrometry
HPLC	high-performance liquid chromatography
HS-SPME-GC-MS	headspace solid-phase microextraction coupled with gas chromatography-mass spectrometry
iTRAQ	Isobaric Tags for Relative and Absolute Quantitation
LC-MS/MS	liquid chromatography-tandem mass spectrometry
LC-MS	liquid chromatography mass spectrometry
LC-QTRAP-MS/MS	liquid chromatography—hybrid triple quadrupole linear ion trap mass spectrometry
LDI-MS	laser desorption/ionization mass spectrometry
MALDI-TOF MS	Matrix-Assisted Laser Desorption/Ionization Time-of-Flight Mass Spectrometry
MS	mass spectrometry
NELDI MS	nanoparticle-enhanced laser desorption ionization mass spectrometry
NMR	nuclear magnetic resonance
N-NOSE	Nematode Nose
Q-ICP-MS	Inductively Coupled Plasma-Mass Spectrometer
qMSP	quantitative methylation-specific PCR
qRT-PCR	quantitative reverse transcription PCR
RIA	radioimmunoassay
RPLC	Reverse-Phase liquid chromatography
SALDI MS	surface-assisted laser desorption/ionization mass spectrometry
SBSE-TD-GC/MS	stir-bar sorptive extraction coupled with thermal desorption gas chromatography/mass spectrometry
SERS	surface-enhanced Raman spectroscopy
SIFT-MS	Selected Ion Flow Tube Mass Spectrometry
SPME	solid-phase microextraction
SPRi	surface plasmon resonance imaging
UHPLC-MS	ultra-high-performance liquid chromatography-tandem mass spectrometry
UPLC-Q-TOF-MS	ultra-performance liquid chromatography quadrupole time-of-flight mass spectrometry
UPLC-MS	ultra-performance liquid chromatography-mass spectrometry
